# Achieving health equity in US suicides: a narrative review and commentary

**DOI:** 10.1186/s12889-022-13596-w

**Published:** 2022-07-15

**Authors:** Seth W. Perry, Jacob C. Rainey, Stephen Allison, Tarun Bastiampillai, Ma-Li Wong, Julio Licinio, Steven S. Sharfstein, Holly C. Wilcox

**Affiliations:** 1 Department of Psychiatry and Behavioral Sciences, College of Medicine, State University of New York (SUNY, Upstate Medical University, Syracuse, NY USA; 2 Department of Neuroscience & Physiology, College of Medicine, State University of New York (SUNY, Upstate Medical University, Syracuse, NY USA; 3 Department of Neurosurgery, College of Medicine, State University of New York (SUNY, Upstate Medical University, Syracuse, NY USA; 4 Department of Public Health and Preventive Medicine, College of Medicine, State University of New York (SUNY, Upstate Medical University, Syracuse, NY USA; 5 Department of Mental Health, Johns Hopkins School of Public Health, Baltimore, MD USA; 6 Department of Psychiatry, College of Medicine and Public Health, Flinders University, Adelaide, Australia; 7 Mind and Brain Theme, South Australian Health and Medical Research Institute (SAHMRI), Adelaide, Australia; 8 Department of Psychiatry, Monash University, Clayton, Australia; 9 Department of Medicine, College of Medicine, State University of New York (SUNY, Upstate Medical University, Syracuse, NY USA; 10 Department of Pharmacology, College of Medicine, State University of New York (SUNY, Upstate Medical University, Syracuse, NY USA; 11 Sheppard Pratt Health System, Baltimore, MD USA; 12 Department of Psychiatry, University of Maryland School of Medicine, Baltimore, MD USA; 13 Department of Psychiatry and Behavioral Sciences, Johns Hopkins School of Medicine, Baltimore, MD USA

**Keywords:** Suicide, Health equity, Health disparities, Depression, Guns, Firearms, Lethal means, Rural, Urban, Geography, Disparity, Public health

## Abstract

Suicide rates in the United States (US) reached a peak in 2018 and declined in 2019 and 2020, with substantial and often growing disparities by age, sex, race/ethnicity, geography, veteran status, sexual minority status, socioeconomic status, and method employed (means disparity). In this narrative review and commentary, we highlight these many disparities in US suicide deaths, then examine the possible causes and potential solutions, with the overarching goal of reducing suicide death disparities to achieve health equity.

The data implicate untreated, undertreated, or unidentified depression or other mental illness, and access to firearms, as two modifiable risk factors for suicide across all groups. The data also reveal firearm suicides increasing sharply and linearly with increasing county rurality, while suicide rates by falls (e.g., from tall structures) decrease linearly by increasing rurality, and suicide rates by other means remain fairly constant regardless of relative county urbanization. In addition, for all geographies, gun suicides are significantly higher in males than females, and highest in ages 51–85 + years old for both sexes. Of all US suicides from 1999–2019, 55% of male suicides and 29% of female suicides were by gun in metropolitan (metro) areas, versus 65% (Male) and 42% (Female) suicides by gun in non-metro areas. Guns accounted for 89% of suicides in non-metro males aged 71–85 + years old. Guns (i.e., employment of more lethal means) are also thought to be a major reason why males have, on average, 2–4 times higher suicide rates than women, despite having only 1/4—1/2 as many suicide attempts as women. Overall the literature and data strongly implicate firearm access as a risk factor for suicide across all populations, and even more so for male, rural, and older populations.

To achieve the most significant results in suicide prevention across all groups, we need 1) more emphasis on policies and universal programs to reduce suicidal behaviors, and 2) enhanced population-based strategies for ameliorating the two most prominent modifiable targets for suicide prevention: depression and firearms.

## Background

Data suggests that the existence of more evidence-based mental health treatments has not significantly reduced depression prevalence and suicide in the US, and that significant personal (i.e., stigma) or practical/logistical barriers to effective mental health care remain [1]. Depression and suicide rates have risen significantly over this same time period, with health disparities identified in both depression and suicide based on age, sex, race/ethnicity, geography, veteran status, sexual minority status, and/or socioeconomic status [2]. Combating these distressing trends to achieve health equity will require greater attention to proactive, evidence-based, sustainable, and practical solutions that address the varied causes, sociodemographics, mechanisms, and differential risk factors of suicide deaths. However, to make progress in these areas, first we must understand precisely and granularly 1) What are the disparities that exist in suicide deaths, and 2) What are the key forces that drive suicides and suicide disparities? In addition, gaining better understanding of the varied (e.g., biologic, sociodemographic, or perhaps even genetic or epigenetic) factors that may promote lower suicide deaths in some populations has great potential to help guide improved prevention strategies for higher-risk populations.

To achieve these goals, this paper will explore sociodemographic disparities that exist in suicide deaths, with emphasis on two of the most significant modifiable targets for suicide prevention: 1) untreated or undertreated depression, and 2) access to the lethal means (firearms) that cause more suicide deaths than all other means combined and thus pose the greatest threat to individual and public health. Furthermore, herein we newly define increased or unsafe (i.e., disparate) access to firearms as a suicide health disparity that promotes health inequities. Finally, we discuss strategies for improving health equity surrounding suicide deaths in each of the areas discussed. Overall, the data suggest that more effective prevention, early identification, and treatment of depression and other mental health disorders that carry suicide risk (e.g., bipolar disorder, schizophrenia), as well as strategically and effectively employed firearm safety measures, would help reduce suicide deaths and improve health equity.

Readers should note that this is not a systematic review, and therefore should not be interpreted as such. Rather, this paper is part narrative review and part commentary, with accompanying presentation of publicly available suicide data to help illustrate our points. By design, this manuscript does not fit neatly into any of the usual boxes, with the intention of providing a unique contribution to the suicide literature. To our knowledge, few previous papers have discussed the health equity aspects of suicide in exactly the same manner or depth as we do here. Second, herein we frame suicide prevention in general, and disparate access to firearms in particular, as public health crises that we propose can be most effectively addressed as health equity/health disparity issues. Third, we present recent and historical National Center for Health Statistics (NCHS) mortality and morbidity data (CDC Wonder database; [3]), bolstered by support from the literature, to illustrate disparities in the suicide domain pointing to the need for enhanced population-based strategies for ameliorating the two most prominent modifiable targets for suicide prevention: depression and firearms. We hope these perspectives will stimulate useful new discussions, insights, and research into these pressing public health concerns.

## Main text

### Suicide disparities as a health equity concern: a new framework?

The *National Stakeholder Strategy for Achieving Health Equity* defines health equity as the “attainment of the highest level of health for all people. Achieving health equity requires valuing everyone equally with focused and ongoing societal efforts to address avoidable inequalities, historical and contemporary injustices, and the elimination of health and healthcare disparities” (page 9 in [4]). These principles can and should be applied to reducing suicide deaths, while we still hope and strive for the utopian goals of no suicide (or disease-related) deaths. Suicide is linked with social determinants of health and healthcare disparities based on age, sex, race/ethnicity, socioeconomic status, geography/urbanization, veteran status, sexual minority status, and access to firearms, among others.

In this data review and commentary we propose that these many disparities in suicide rates, from differences linked to sex, race, age, socioeconomic status, or any other factors – including disparate access to firearms – can and should all be viewed and addressed as health equity issues. We aim to expand and integrate previous discussions of health disparities in suicide to stimulate new insights for addressing this growing public health crisis. We suggest that it is through this "health equity" lens by which we may best desensitize and depoliticize these complex and often discomforting divisive topics to maximize society's embrace of shared goals and meaningful progress in suicide prevention at all levels, from legislative action to the dissemination and implementation of universal programs aimed at the general public.

### Suicide and health disparities

The Centers for Disease Control and Prevention (CDC) data reveal that the overall age-adjusted US suicide rate increased by 35% from 1999—2018 (10.5 to 14.2 per 100,000 standard population) [5, 6] followed by a decrease in 2019 [7], with notable disparities based on sex, age, race/ethnicity, income, education, and geography (urbanization), among others [5, 8] (and see this section). This has led the CDC to call for acceleration of “efforts to eliminate health disparities [in suicide] with a focus on surveillance, analysis, and reporting of disparities and the identification and application of evidence-based strategies to achieve health equity” [9]. Accordingly, in this section we will highlight suicide disparities interrogated from the publicly available National Center for Health Statistics Mortality Data from CDC WONDER (https://wonder.cdc.gov/ucd-icd10.html). A detailed description of this dataset is here (https://wonder.cdc.gov/wonder/help/ucd.html). Although this is a foundational dataset from which much reporting of US suicide data is done for both scientific and lay publications, it is informative to note that commonly captured and reported suicide data may in fact under-report self-injury mortality, particularly for some populations [10–12]. For footnote, see [13].

#### Sex

From 1999–2019, the age-adjusted suicide rate for females (all ages and races/ethnicities) increased from 4.0 to 6.0 per 100,000 population (a 50% increase), whereas the age-adjusted male suicide rate increased from 17.8 to 22.4 per 100,000 (a 26% increase) [3]. Therefore, while males experienced a lesser rise in suicide rates over this period, their suicide rates were 4.5 times those of females in 1999 and 3.7 times those of females in 2019. There is a recent narrowing of the gender gap in suicide.

#### Age

**Fig. 1:**
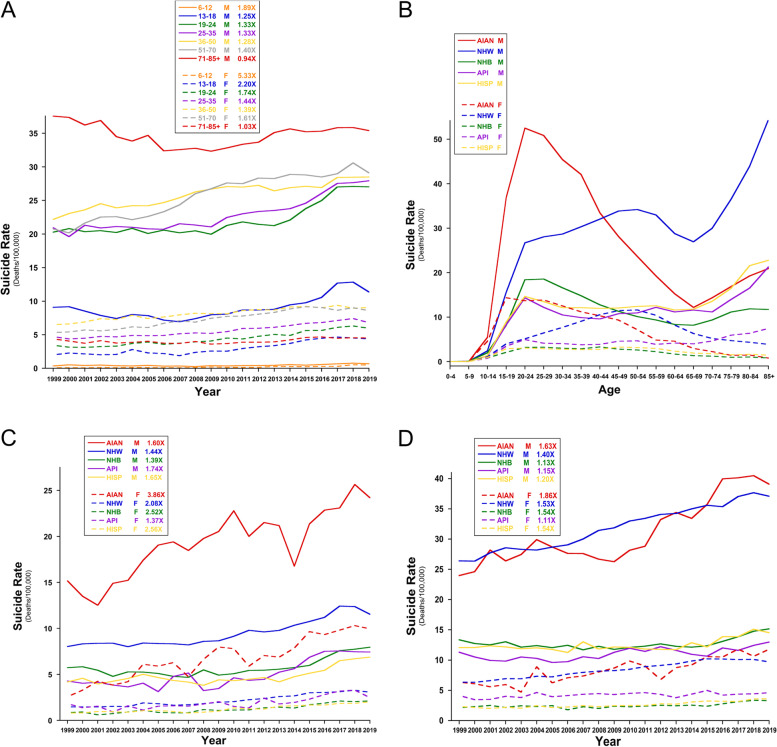
Suicide Disparities by Age, Race/Ethnicity, and Sex. Suicide deaths per 100,000 are shown by **A** Age bracket and year, **B** Age bracket and race/ethnicity over the lifespan using cumulative 1999–2019 data, **C** 24 yo and under by race/ethnicity and year, and **D** Over 24 yo by race/ethnicity and year. All rates are stratified by age and are crude rates for males (M) and females (F) separately. All data is from CDC WONDER Underlying Cause of Death [3]

In males, suicide rates generally increase with age and are highest in the ≥ 71 years old (yo) age bracket, followed by 51–70, 36–50, 25–35, 19–24, 13–18, and 6–12 yo age brackets (Fig. 1A). In recent years the differences in suicide rates between younger and older males have narrowed, as younger male suicides have increased sharply since 2009. In the ≥ 71 yo group, suicide rates declined slightly from 1999–2019 (0.94x), although they have increased steadily from a low point in 2009. In all other male age brackets suicide rates have increased since 1999, and typically most sharply since about 2005–2008, with 6–12 yo males seeing the largest increase from 1999–2019 (1.89x), followed by 51–70 yo males (1.4x). In females, 1999–2019 suicide rates have been highest in 36–50 yo, followed closely by 51–70 yo, then 25–35, 19–24, 71–85 + , 13–18, and 6–12 yo (Fig. 1A). From 1999–2019, female suicide rates rose in all age groups except 71–85 + yo, which remained steady (1.03x). The greatest fold-increases were observed in 6–12 yo (5.33x), followed by 13–18 (2.20x), 19–24 (1.74x), 51–70 (1.61x), 25–35 (1.44x), and 36–50 (1.39x) yo. Thus young females ≤ 24 yo have the most rapidly rising suicide rates since 1999, with suicide rates for 13–18 and 19–24 yo females now equaling or surpassing, respectively, rates for 71–85 + yo females (Fig. 1A). As with males, most female age brackets’ sharpest rise has generally occurred since ~ 2005–2008.

#### Race/ethnicity stratification reveals hidden patterns

In the general population, when looking at suicide rates across the lifespan as above, suicide rates peak in mid-life for women and late-life for men (Fig. 1A). However, these numbers are skewed toward those of the non-Hispanic white (NHW) population, which comprises the greatest numbers of individuals and suicide deaths. When the numbers are further stratified for race/ethnicity, different patterns are revealed that could inform approaches for demographically-targeted suicide prevention and outreach.

Suicide deaths for NHW females indeed increase steadily to a mid-life peak at about ages 45–54, followed by tapering into older age (Fig. 1B). However, in Hispanic (HISP) and non-Hispanic black (NHB) females, rates reach an initial peak at ages 15–19 and 20–24 respectively, followed by a fairly steady plateau until about age 50, then a steady decline thereafter. Asian/Pacific Islander (API) females also show this early peak-then-plateau pattern, except unlike HISP and NHB (and NHW) females whose suicide rates decline after middle age, API suicide rates then sharply increase from about age 50–55 onward (Fig. 1B). That makes API females the only female racial/ethnic group with increased suicide rates beyond age 50, a pattern more commonly seen in older males (next paragraph). Finally, suicide rates in American Indian/Alaska Native (AIAN) females sharply increase to peak at 15–19 yo, followed by a gradual but steady decline into older adulthood. Together these data indicate that among females, AIANs have the highest suicide rates until about age 40; NHW females have the highest rates from ages 40–70, and API females have the highest rates after age 70. Identifying the factors that may make some racial/ethnic groups more or less vulnerable to suicide during certain life stages (ages) is worth further exploration to improve targeted suicide prevention efforts.

In males, suicide rates across the lifespan amongst different racial/ethnic groups are generally more similar to one another (Fig. 1B). Suicide rates in all male racial/ethnic groups increase sharply to age 20–24, with AIAN male suicide rates far surpassing those of all other groups at this age bracket. From age 20–24 onward, AIAN and NHB male rates decline steadily to age 65, then increase thereafter. HISP and API male rates are fairly steady until age 65 or 70 respectively, then increase after that. NHW male suicide rates rise from age 20–24 to a second peak at age 50–54, decline to ages 65–69, then increase the most sharply after that. Comparing across all male racial/ethnic groups, AIAN males have the highest suicide rates until age 40–44, whereas NHW males have the highest rates after age 40–44 (Fig. 1B).

Thus in males, all racial/ethnic groups experience sharply increasing suicide rates to age 20–24 and after ages 65–70. In females, the age of the first peak (or plateau) in suicide rates is more variable by racial/ethnic group, and after ages 65–70, female suicide rates decline across all racial/ethnic groups except API (which, like males, also increase in late age). Finally, while female suicide rates are on average lower than male suicide rates, the suicide rates of some female populations may surpass those of some male populations at several points in the lifespan, particularly for younger AIAN females and middle-aged NHW females (Fig. 1B).

Readers can refer to several other excellent reports for additional detailed discussion of racial/ethnic disparities in US suicides [14–18].

#### Youth versus adult suicides

For both males and females, the sharp rise in suicide rates since about 2007 is particularly pronounced in youth and young adults (≤ 24 yo), and especially young females (Fig. 1C), compared to older adults (≥ 25 yo) (Fig. 1D). This trend has been highlighted elsewhere [19]. All ≤ 24 yo male and female populations experienced a rise in suicide rates between 1999 and 2019, ranging from 1.39 (NHB) to 1.74 (API) fold increases for males, and 1.37 (API) to 3.86 (AIAN) fold increases for females (Fig. 1C). Differences in suicide rates across ≤ 24 yo female populations have remained relatively constant, except for ≤ 24 yo AIAN females, whose rates have increased disproportionately to all other racial/ethnic groups. This ≤ 24 yo AIAN female group now has higher suicide rates than all ≤ 24 yo male populations except NHW and AIAN (Fig. 1C). Likewise, since 1999, there are increasing disparities in the suicide rates of ≤ 24 yo NHW and especially AIAN males compared to other same-age male populations (Fig. 1C).

In contrast, among ≥ 24 yo males, AIAN experienced the greatest rise in suicide rates between 1999–2019 (1.63x), followed by NHW (1.40x), HISP (1.20x), API (1.15x), and NHB (1.13x) males, respectively (Fig. 1D). As a result, suicide rate disparities between ≥ 24 yo NHW and AIAN, and other same-age male populations have grown in recent years (Fig. 1D). Among ≥ 24 yo females, AIAN suicide rates increased the most from 1999–2019 (1.86x), followed by NHB and HISP (both 1.54x), then NHW (1.53x), and API (1.11x) females, respectively. As with ≥ 24 yo males, NHW and AIAN female suicide rates have grown disproportionately to other ≥ 24 yo female racial/ethnic groups. For both age groups and all racial/ethnic groups, except API, females had equal or most often greater rises in suicide rates versus their male counterparts.

Together these data highlight many disparities in suicide rates across age, sex, and racial/ethnic groups. A better understanding of the factors driving these disparities is needed to achieve more effective suicide prevention efforts.

#### Sexual or gender minority status

Data suggest that those having a minority sexual orientation or gender identity are at increased risk for suicide across ages and race/ethnicities [20–29], and in other groups at high risk for suicide such as veterans [30, 31]. At present, though, the numbers of individuals in these groups who die by suicide are difficult to track because sexual orientation and gender identity information is typically not collected at time-of-death [32]. These are critical areas for further research, improved data collection, and targeted suicide prevention efforts.

Current research indicates that the risk of suicide may vary among subgroups of sexual minorities. Although a systematic review on suicidal thoughts and behaviors found mixed results regarding differences between bisexual and gay or lesbian individuals [33], two recent meta-analyses suggest that bisexual individuals may be at a higher risk of suicide attempts than gay or lesbian individuals [22, 34]. Unfortunately, suicide research on sexual minorities that do not identify as gay, lesbian, or bisexual is sparse. However, a recent study of college students found that those identifying as pansexual, bisexual, queer, or primarily gay or lesbian had higher odds of endorsing two or more risk factors for suicide (i.e., depression, alcohol misuse, suicidal ideation, or suicide attempt) compared to mostly heterosexual, gay or lesbian, asexual, or other sexual minority [35]. As a result, more studies are warranted among subgroups of sexual minorities that are traditionally not distinguished in suicide research. Likewise, more suicide research is needed among gender minorities, since few studies examine whether subgroups within gender minorities (e.g., transgender, nonbinary, Two-Spirit) may differ from one another in terms of suicide.

#### Veterans

America's veteran population is also at increased risk for suicide. From 2005 to 2017, age- and sex-adjusted suicide rates increased 22% in the general population (from 14.7 to 18.0 per 100,000) but increased by 50% amongst veterans over the same time period (from 18.5 to 27.7 per 100,000) [36]. In 2017, veterans' suicide rate was 1.5 × the rate of non-veteran adults (using age- and sex-adjusted rates) [36]. The US Department of Veterans Affairs (VA) and US Department of Defense have recently updated and released comprehensive guidelines for assessing and managing veteran and military populations at risk for suicide [37, 38]. These guidelines and materials are notable for their common-sense, empowering, and collaborative language around the issue of lethal means risk assessment and reduction in at-risk veterans, including but not limited to firearms, and as such may provide a useful model for broader national conversations around these issues as they might be applied to suicide prevention in the civilian population. That a substantial number of veterans may not utilize or be eligible for VA care only highlights the urgency and need to translate programs of this nature to the broader US population [39]. Reports indicate that 70 percent of veterans who died by suicide were not engaged with VA care or services in the two years before their death [40–42]. The recent (March 5, 2019) President’s Roadmap to Empower Veterans and End a National Tragedy of Suicide (PREVENTS) executive order [43] and other similar state and local initiatives [36, 42] intended to increase resources and efforts devoted to reducing suicides of veterans and other service members, especially outside the VA network, are important steps that may have useful translations to the broader public.

#### Geography (county urbanization/rurality)

**Fig. 2:**
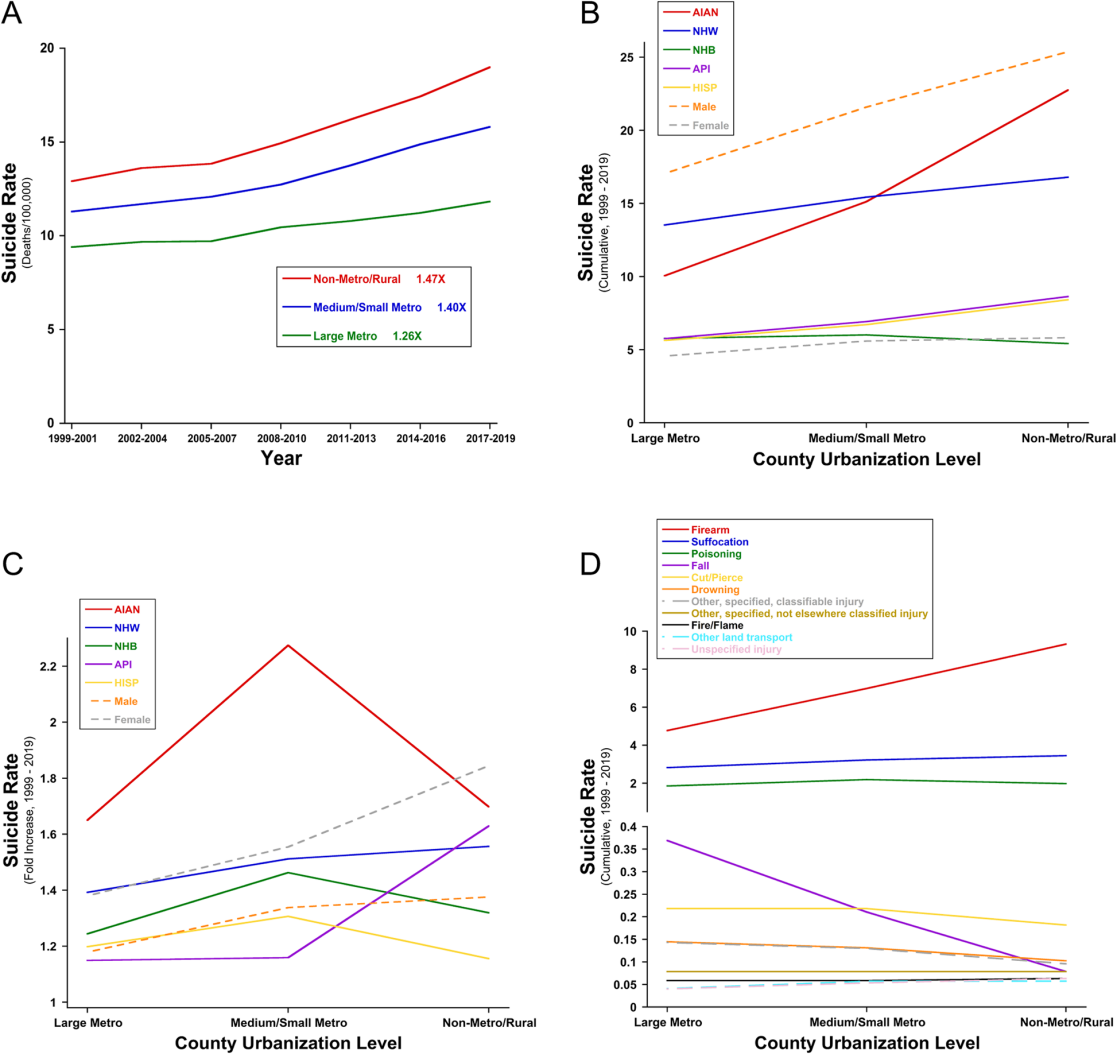
Suicide Disparities by Urbanization, Race/Ethnicity, Sex, and Mechanism of Death. Suicide deaths per 100,000 by **A** Year and relative county urbanization, **B** County urbanization, race/ethnicity, and sex (aggregate 1999–2019), **C** Fold-increase in suicide 1999–2019, by race/ethnicity and sex, and **D** County urbanization and mechanism of death (aggregate 1999–2019). All age-adjusted, 2000 US population. 2006 county urbanization classification [48]. All plotted data are derived from CDC WONDER Underlying Cause of Death [3]

Within the US, suicide rates are consistently highest in rural areas, followed by medium-sized cities, and lowest in large cities (Fig. 2A) [3, 8, 44–47]. This observation is valid for both men and women, and all racial/ethnic groups, except for NHB (Fig. 2B) (and see below) [3, 8]. In other words, increased suicide rates track closely with increasing county rurality (decreasing urbanization). From 1999–2019, suicide rates increased in all county urbanization categories but increased more rapidly in medium/small metro and non-metro/rural areas [3, 8, 45], resulting in growing disparities in suicide rates between these regions (Fig. 2A). Strikingly, all racial/ethnic groups, all age groups, and both men and women experience their highest suicide rates in rural areas and lowest suicide rates in large metro areas, except NHB who experience slightly lower suicide rates in the most rural versus the most urban areas (Fig. 2B) [3, 8]. Moreover, for both males and females (all races/ethnicities combined), the rise (change) in suicide rates from 1999 to 2019 was greatest in the most rural (least urban) counties, and smallest in the least rural (most urban) counties (Fig. 2C). For example, from 1999 to 2019, male suicide rates increased 1.18 × in large metro counties, 1.34 × in medium/small metro counties, and 1.38 × in non-metro/rural counties. Similarly but even more pronounced, from 1999 to 2019, female suicide rates increased 1.38 × in large metro counties, 1.55 × in medium/small metro counties, and 1.84 × in non-metro/rural counties.

Data stratified by race/ethnicity (both sexes combined) show that some groups experienced their greatest rise in 1999–2019 suicide rates in non-metro/rural counties (i.e. the most rural areas) (NHW, API), whereas other groups experienced their greatest rise in 1999–2019 suicide rates in medium/small metro counties (i.e. moderately rural areas) (AIAN, NHB, HISP). However, all groups except HISP experienced their *lowest* rise in 1999–2019 suicide rates in the most urban (least rural) counties (Fig. 2C).

Table 1 shows that in 1999, overall suicide rates were 10.04 (per 100,000) in metro areas and 12.56 in non-metro areas, a 1.251-fold difference. In 2019, overall rates were 13.19 in metro and 18.90 in non-metro areas, a 1.434-fold difference. Furthermore, from 1999 to 2019, the overall suicide rate increased 1.313 × in metro areas (10.04 to 13.19/100,000) and 1.505 × in non-metro areas (12.56 to 18.90/100,000). Together these data highlight growing disparities in suicide rates by relative degree of urbanization, that are consistent across sex and race/ethnicity.

**Table 1:** All Suicides, 1999 versus 2019, by County Urbanization

	**1999**	**2019**	**Raw Increase**	**Fold Increase**
**Metro**	10.04	13.19	3.14	1.313
**Non-Metro**	12.56	18.90	6.34	1.505
**Raw Increase**	2.52	5.72		
**Fold Increase**	1.251	1.434		

All data is age-adjusted deaths per 100,000 (2000 US population) [3], except Fold Increase. 2006 county urbanization classification [48]

#### Suicide means versus county urbanization/rurality

Relative urbanization/rurality also creates a means disparity, with regard to the mechanism of death by which suicides occur. Cumulative suicide rates 1999–2019 demonstrate that suicide means were relatively consistent across the different urbanization geographies, with two exceptions: firearms and falls (from height). The rate of firearm suicides approximately doubled, in a very linear fashion, as relative urbanization decreased, and suicides from falls approximately quintupled linearly as relative urbanization increased (Fig. 2D). Together these data implicate means availability as a likely risk factor for suicide, at least as regards firearms (more prevalent in rural areas) and falls from tall structures (less prevalent in rural areas). Moreover, using cumulative suicide data from 1999–2019 [3], both sexes, and all racial/ethnic and age groups examined, had higher gun suicide rates and higher percentages of suicides by gun in non-metro versus metro areas (Table 2). The only exceptions were NHB females, who had equivalent gun suicide rates in metro versus non-metro areas (both 0.59), and AIAN females, who had an essentially equivalent percentage of suicides by gun in metro versus non-metro areas (23.5% versus 23.4%, respectively) (Table 2). The implications of these observations for health equity are discussed in greater detail below.

**Table 2:** Gun Suicides, 1999 to 2019, by County Urbanization, Sex, Age, and Race/Ethnicity

		METRO			NON METRO	
Population	Gun Suicides	All Suicides	% Gun Suicides	Gun Suicides	All Suicides	% Gun Suicides
6–12 yo M	0.10	0.43	22.4	0.21	0.56	37.8
13–18 yo M	3.87	8.18	47.3	7.57	12.77	59.3
19–24 yo M	10.51	20.72	50.7	17.41	29.01	60.0
25–35 yo M	9.98	21.35	46.8	17.83	32.70	54.5
36–50 yo M	11.34	24.32	46.6	19.77	33.66	58.7
51–70 yo M	14.86	25.21	58.9	22.27	30.41	73.2
71–85 + yo M	25.41	32.81	77.5	37.38	42.16	88.7
AIAN M	8.29	19.63	42.2	16.26	36.22	44.9
NHW M	12.77	22.85	55.9	18.37	27.68	66.4
NHB M	5.67	10.22	55.5	5.94	9.25	64.2
API M	2.50	8.87	28.2	4.47	13.50	33.1
HISP M	4.11	9.85	41.7	7.07	13.55	52.1
All Males	10.19	18.63	54.7	16.58	25.37	65.4
6–12 yo F	0.02	0.18	10.6	*0.04*	0.21	17.2
13–18 yo F	0.63	2.85	22.2	1.13	3.62	31.0
19–24 yo F	1.23	4.33	28.3	1.93	5.00	38.6
25–35 yo F	1.61	5.41	29.9	2.88	7.22	39.9
36–50 yo F	2.24	7.78	28.8	4.10	9.92	41.3
51–70 yo F	2.27	7.47	30.4	3.64	7.64	47.7
71–85 + yo F	1.33	4.23	31.3	1.80	3.47	51.8
AIAN F	1.46	6.21	23.5	2.27	9.72	23.4
NHW F	1.94	6.28	30.8	2.77	6.34	43.7
NHB F	0.59	2.08	28.3	0.59	1.54	38.1
API F	0.34	3.44	9.8	0.92	4.33	21.1
HISP F	0.45	2.16	20.8	0.71	2.53	28.1
All Females	1.43	4.92	29.1	2.47	5.82	42.3

All data is age adjusted deaths per 100,000 (rate), except % suicides by gun. All data is cumulative 1999 to 2019

*Italics:* Rate is based on 20 or fewer deaths (*n* = 17 deaths in this case) and may be unstable. Interpret with caution

Data source: https://wonder.cdc.gov/ucd-icd10.html

#### Socioeconomic status/poverty

Another recent study found that in children aged 5–19 years, suicide risk and gun suicide risk were increased 37% and 87% respectively between the least and most impoverished counties, even after controlling for county urbanicity [49]. This work again confirmed significantly increased rural (1.66x) versus urban suicide rates over the study period and population, with no collinearity between independent variables [49]. This study also identified a means disparity, as we report here (Fig. 2D and discussed above), with suicides by firearms associated with county poverty level, whereas suicides by suffocation or poisoning showed no association with poverty [49]. Future research should explore whether county poverty and rurality may be independent risk factors for suicide and firearm suicide, the nature of this relationship (e.g., perhaps suicide is driven by economic distress in poverty, and social isolation or other factors in the case of rurality), and whether poverty and rurality suicide risks synergize when found in combination.

Some findings of associations between socioeconomic status and suicide risk have been variable depending on study design. Moreover, higher socioeconomic status does not universally protect against suicide risk at the individual level, especially when acute or chronic mental distress exists, as the numerous higher-profile suicides of celebrities and public figures in recent years have unfortunately made all too clear. Nonetheless, many recent and comprehensive studies of aggregate suicide risks have identified associations between lower socioeconomic status or higher poverty and increased suicide risk among children and adults, in the US and elsewhere [49–55]. However, associations between socioeconomic status and suicide risk may vary depending on age or race/ethnicity [56], as well as study design (e.g., whether aggregate or individual or instantaneous risk is considered), suggesting that the relationships between suicide risk and socioeconomic status are likely complex and multifactorial, and require further investigation. However, on the whole, US suicide rates reflect significant disparities based on socioeconomic factors such as income or education, with higher suicide rates typically associated with lower socioeconomic status, income, and/or education. In the "Strengthen Economic Supports" section of "Preventing Suicide: A Technical Package of Policies, Programs, and Practices," the CDC lists "strengthening household financial security" and "housing stabilization policies" as two strategies evidenced as being effective for reducing suicide risk associated with economic stress [57]. In this light, it would be worthwhile to investigate whether disparate availability of federal, state, and local COVID-19 economic support programs might help explain why some but not all groups experienced reduced suicide rates during the COVID-19 pandemic [58].

#### Section summary

Together these data on suicide health disparities yield several new or perhaps underappreciated insights. Although males have on average about 3.7 × higher suicide rates than females (as of 2019 data), this gender gap is narrowing because female suicides are increasing at a higher rate. Suicides in NHW, API, and HISP males trend upward with increasing age, but peak at younger ages and then trend downward with age in AIAN and NHB males. Likewise, suicides in AIAN females also peak in the teens and early twenties and then decline with age thereafter. API females are the only female racial/ethnic group that experiences rising suicides after age 65, which makes them more similar to men in that regard; all male racial/ethnic groups experience pronounced and steady rises in suicide rates after age 65. Year-over-year suicide rates are rising especially fast in children and young adults aged ≤ 24, compared to adults aged ≥ 25. Individuals who are veterans, or who have a minority sexual orientation or gender identity, are also at higher suicide risk. Lower socioeconomic status is associated with higher suicide risk in both children [49] and adults [50, 51], although socioeconomic disparities in suicide rates may differ further by age or race/ethnicity in some studies [56]. The relationships are complex and multifactorial and require further study to better understand how various demographic factors intersect to impact suicide rates in different populations.

Finally, overall and stratified suicide rates are consistently higher, have been rising faster, and have increased more over time in more rural versus more urban areas, and these trends are consistent across sex and racial/ethnic groups, with very few exceptions (Fig. 2). Furthermore, and perhaps underappreciated, county urbanization/rurality is also linked with two means disparities for suicide deaths: firearm suicides increase linearly with increasing county rurality (Fig. 2D) (or poverty [49]), and suicides from falls decrease linearly with increasing county rurality (Fig. 2D). In contrast, suicide rates by other means remain fairly constant regardless of relative county urbanization (Fig. 2D) (or poverty [49]). Gun suicide rates and percent suicides by gun are significantly higher in rural versus urban counties (Table 2), as well as in more impoverished counties in children even when controlling for rurality [49] (adults were not investigated in this latter study). Future work must clarify the underlying mechanisms that create these many health disparities in suicide deaths, so that we may develop more targeted and effective suicide prevention efforts.

### What drives these disparities?

#### A) Depression

Depression is a leading psychiatric risk factor for suicide, with ~ 60% of suicides occurring among those with mood disorders [59]. However, most individuals who suffer from depression do not make an attempt or die by suicide: roughly 2% of those with a history of outpatient depression treatment will die by suicide, versus 4% of those with a history of inpatient depression treatment [59]. Approximately 7% of men and 1% of women with lifetime histories of depression will die by suicide [59]. While having a diagnosis of any mental health disorder increases suicide risk [60], a meta-review analysis of suicide mortality in mental health disorders revealed that only borderline personality disorder (BPD) – which often co-occurs with depressive disorders – carried a higher suicide risk than depression (Table 3) [61].

**Table 3:** All-cause and suicide mortality in mental health disorders

Diagnosis	All-cause mortality risk estimate (95% CI)	Statistic	Men (95% CI)	Women (95% CI)	Suicide risk estimate (95% CI)	Statistic	Men (95% CI)	Women (95% CI)	AMSTAR score
Opioid use (6,14)	14.7 (12.8-16.5)	SMR			13.5 (10.5-17.2)	SMR	7.6 (4.4-12.1)	3.6 (0.1-19.9)	7, 1
Amphetamine use (15)	6.2 (4.6-8.3)	SMR*	5.9 (4.1-8.1)					31.0 (21.0-44.0)	8
Cocaine use (16)	4 to 8	SMRs							7
Anorexia nervosa (17,18)	5.9 (4.2-8.3)	SMR				SMR*			2, 3
Alcohol use disorder (19,20)	4.6 (2.7-7.7)	RR	3.4 (3.0-3.8)			SMR	8.8 (6.4-12.1)	16.4 (10.7-25.2)	5, 5
Autism spectrum disorder (21)	2.8 (1.8-4.2)	SMR	2.1 (1.7-2.7)						7
**Heavy smoking** (22)		**RR - WA**	**2.4**	**2.7**					**2**
Schizophrenia (1)	2.5 (2.2-2.4)	SMR	3.0	2.4	12.9 (0.7-174.3)**	SMR*			6
Dementia (23)	1.5 to 3.0	RRs							5
Moderate smoking (22,24)		RR -WA	2.0	2.0	1.8 (1.5-2.2)	RR	1.7 (1.4-2.1)	1.8 (1.2-2.7)	2, 6
Bulimia nervosa (17,18)	1.9 (1.4-2.6)	SMR				SMR*		7.5 (1.6-11.6)	2, 3
Eating disorder NOS (17)	1.9 (1.5-2.5)	SMR							2
Depression (25,26)	1.6 (1.6-1.7)	RR			19.7 (12.2-32.0)	SMR			7, 3
Depression in the elderly (27)	1.6 (1.4-1.8)	RR							4
Dysthymic disorder (27)	1.4 (0.9-2.0)	RR							4
Cannabis use (28)		RRs	1.2 to 1.3	1.1 (0.8-1.5)					4
Borderline personality disorder (29)					45.1 (29.0-61.3)	SMR*			1
Bipolar disorder (26)					17.1 (9.8-29.5)	SMR			3
Personality disorders (30)						RR	4.1 (3.0-5.8)	1.8 (0.7-5.2)	3
Anxiety disorder (any type) (31)					3.3 (2.1-5.3)	OR			7
Post-traumatic stress disorder (31)					2.5 (0.5-13.4)	OR			7

SMR – standardized mortality ratio, OR – odds ratio, RR – relative risk, WA – weighted average, AMSTAR – Assessing the Methodological Quality of Systematic Reviews, NOS – not otherwise specified

*Not adjusted for random effects, **90% confidence intervals

Table 3 is reprinted from [61] with permission

Unfortunately, many of those with depression are not treated, undertreated, or unsuccessfully treated, likely contributing to these statistics. The following sentences are noncontiguous excerpts from [62], with some paraphrasing but most text verbatim:

"Among adolescents aged 12 to 17 who had a past year MDE with severe impairment, receipt of treatment for depression in the past year was 49.7 percent (or 1.3 million people) in 2019. Among the 19.4 million adults aged 18 or older in 2019 who had a past year MDE, 66.3 percent (or 12.8 million people) received treatment for depression in the past year. Among adults aged 18 or older in 2019 who had past year mental illness and a perceived unmet need for mental health services but did not receive services in the past year, the most common reason for not receiving services was they could not afford the cost of care (43.9 percent for these adults with any mental illness (AMI) and 51.8 percent for these adults with serious mental illness(SMI)). Other common reasons for not receiving services included not knowing where to go for services (33.1 percent for these adults with AMI and 36.8 percent for these adults with SMI) and believing they could handle the problem without treatment (30.5 percent for these adults with AMI and 27.3 percent for these adults with SMI). In addition, 23.4 percent of these adults with SMI were concerned about being committed to a psychiatric hospital or having to take medication "[62].

Are US depression rates or disparities correlated with US suicide rates or disparities? It appears they may be, to some extent. A synthesis of National Survey on Drug Use and Health (NSDUH) data, comprised of 607,520 US individuals aged 12 and older, found that the prevalence of those having a past-year major depressive episode (PYMD) increased significantly in the US from 2005–2015 [63]. Upon stratifying by and controlling for various demographics, this survey identified significant increases and disparities in PYMD over the same time period based upon: 1) Sex: PYMD was significantly increased in both males and females; 2) Age: PYMD was significantly increased only in 12–17, 18–25, and 50 + yo, but not 26–34 and 35–49 yo; 3) Race/Ethnicity: PYMD was significantly increased in NHW, but not in NHB or HISP [the NSDUH "all other races" category, which included AIAN and API, trended toward increased past-year depression rates in adjusted analysis (*p* < 0.0553) and was significant in unadjusted analysis (*p* < 0.0130)]; 4) Annual Family Income: PYMD was significantly increased for < $20,000/year and ≥ $75,000/year, but not for $20,000—$49,999/year (and for $50,000—$74,999/year by unadjusted analysis only); 5) Education Level: PYMD was significantly increased from 2005–2015 for those with some college, and for college graduates, but not for those with some high school, or for high school graduates; all results reported here reflect adjusted analyses, unless noted [63]. Moreover, the increase of PYMD was significantly greater for 12–17 yo versus all other age groups. (See reference [62] for a description of NSDUH survey methods. Even though the NSDUH survey methods are private and confidential, previous research has suggested that some groups may be more or less likely to report depression symptom, even where depression likely exists [64–70]. Thus the possibility of biases by sex, race/ethnicity, socioeconomic status, or other group characteristic must be considered when examining subgroup differences in self-reported depression prevalence.)

As with US suicides, these NSDUH data indicate widespread increases in US PYMD from 2005–2015 in both males and females, with some disparities, and with PYMD rates rising most rapidly in youth and young adults. Compared to the stratified suicide data over this same period (Fig. 1), some observations emerge. First, as with PYMD, the increases in US suicide rates have also been most pronounced in youth versus adults (Fig. 1A). Second, when stratified by race/ethnicity, the groups that had no changes in PYMD from 2005—2015 (NHB and HISP) also generally had more stable suicide rates over this period versus most other racial/ethnic groups (except API) (Fig. 1D). Only NHW experienced increases in both PYMD and suicides from 2005—2015.

The most recent NSDUH data shows PYMD trending upward from 2015–2019 for all age groups except 50 + yo, with the sharpest rises in those aged 12–25 yo (see Figs. 48–50 in [71]). These PYMD curves roughly parallel the changes in suicide rates by age group over the same 2015–2019 time period (compare to Fig. 1B). PYMD also significantly increased between 2015 and 2019 for < 18 yo NHW, NHB, API, and HISP [72], and for ≥ 18 yo NHW, NHB, and HISP [73], again with the sharpest rises in youth as with suicides. The 2015–2019 PYMD trends by race/ethnicity were upward even where they did not reach significance, often due to relatively lower sample numbers [72, 73].

Together these results clarify that while major depression and/or undiagnosed depression are likely to be significant medically-related drivers of suicide deaths, they are by no means the only factor. Other medical (e.g. other mental illnesses; Table 3) and non-medical/sociological (e.g. increased access to firearms, and socioeconomic) risk factors also likely contribute significantly to suicide deaths, as discussed herein.

In total, suicide rates and disparities are increasing, and depression is rising in aggregate and among many demographics which correlate with rising US suicide rates. However, these rising and disparate suicide rates are unlikely to be due simply to higher or differing rates of depression or other diagnosed mental illness [74]. Issues of access to care; identification of latent mental health conditions or other debilitating social and life factors that do not rise to the level of diagnosable mental illnesses; as well as access to disproportionately lethal means, are all factors that we must do better at addressing among all groups, and perhaps particularly among underserved and marginalized populations. These imperatives notwithstanding, the available data clearly illustrate that in order to most effectively minimize suicides and maximize public health in the US, we will need to employ multi-pronged preventive measures that reach well beyond only those individuals with pre-identified mental health issues.

#### B) Sociological driving forces

In two papers that generated vigorous discussion and mild controversy (expertly discussed in [75]), Anne Case and Angus Deaton, the 2015 Nobel Laureate for Economics, highlighted the unprecedented rise in midlife mortality among socioeconomically disadvantaged middle-aged NHW Americans [76, 77]. Those researchers linked rising midlife mortality to a decline in the NHW working class’s economic prospects during an era of rising income inequality in the USA. Widening income inequality was associated with greater health inequality [76, 77], as Americans’ socioeconomic status is directly correlated with longer, happier, and healthier lives [78]. Further, the authors linked increased mortality and disproportionately rising suicide rates among NHW to socioeconomic and educational disparities, chronic ailments, and generational (birth-year) cohort effects, with more recent generations faring worse in most or all categories examined [76, 77]. From this, they postulate a "*cumulative disadvantage* from one birth cohort to the next, in the labor market, in marriage and child outcomes, and in health" [77] as driving increased NHW mortality and suicides (i.e., drug/alcohol-related deaths and suicides, or "deaths of despair"). A detailed and more recent analysis found associations between higher suicide rates and higher: deprivation (especially in rural areas); social fragmentation; percent uninsured; and percent veterans [79]. Lower suicide rates, in contrast, were associated with higher social capital [79]. Others have presented similar or complementary data and analyses, with similar conclusions and useful discussions [80, 81].

Following their examination of the NSDUH data, which showed that compared to other racial/ethnic groups, only NHW experienced a significant increase in depression from 2005–2015, Weinberger et al. presented a thoughtful, comprehensive, and comparative epidemiological discussion of depression risk factors, demographics, and driving forces amongst the racial/ethnic groups surveyed [63] (which closely interrelates with the expert discussion in [75]). [Nonetheless, and particularly in populations or individuals with significant disparities, we cannot discount the possibility that much depression may go undiagnosed, either because it may differ from a classic clinically-diagnosable depression, such as demoralization or disenfranchisement, or it may simply go unsurveyed. In addition, by grouping several races/ethnicities into an "all other races" category, the NSDUH survey may have missed detecting some changes in depression that might have been apparent with further stratification by race/ethnicity into AIAN and API groupings.] Of interest in the context of these analyses, one of the most consistently identified risk factors for depression and suicide, broadly across races/ethnicities and nationalities in the US and worldwide, is one’s income bracket [63, 76, 77, 82], or perhaps more specifically, one’s income “rank” or socioeconomic status (often also correlated with lower educational status) [83–87].

Many AIAN populations may share similar risk factors and disparities – very high poverty rates, low economic prospects, education gaps, frequent rural living, as well as lower access to mental health care and high gun ownership rates – and these same factors may be key contributors to the rise in suicides amongst this population over the same period [88]. Others have also noted the disparate rise in premature mortality among NHW and AIAN, with these increases being “mainly attributable to accidental deaths (primarily drug poisonings), chronic liver disease and cirrhosis, and suicide,” in contrast to declining premature mortality for HISP, NHB, and API [89]. Across all racial/ethnic groups, AIAN have both the fastest rising and highest premature mortality [89], and the highest suicide rates, making them a particularly vulnerable population.

At the same time, AIAN suicide rates are not uniform across all AIAN populations. While AIANs overall and many AIAN subgroups have higher suicide rates than NHW, many other AIAN populations have *lower* suicide rates than NHW living in the same region [88]. AIAN ≥ 45 yo or ≥ 65 yo have lower suicide rates than same-aged NHW in much of the US, and AIAN of all ages in the Eastern US also tend to have lower suicide rates than Eastern NHW [88]. Understanding the driving forces for these vastly differing suicide rates among AIAN sub-populations, and compared to other racial/ethnic groups, will help develop more effective suicide prevention measures and reduce health inequities in these vulnerable populations.

Despite favorable public health gains in some areas and lower suicide rates versus other groups (Fig. 1), NHB have the second-highest premature mortality after AIAN. NHB also have high rates of adult depression relative to other groups [82], often coupled with significant gaps in access to mental health care, and have seen a recent sharp rise (and reversal of trend) in premature mortality across all age groups in those with less than a high school education (similar to NHW in this regard, in recent years) (Fig. 1.2 in [77]). All of which remain significant public health considerations that require remedy.

Particularly against the backdrop of polarizing current events, the topics of race, ethnicity, class, culture, and historical or contemporary discrimination, oppression, or trauma, can be especially sensitive issues that are extraordinarily difficult to adequately and comprehensively address, even in the context of health disparities and when authors have only humanitarian intentions. In this light, the controversy and discussion that emerged from the Case and Deaton papers have been very thoroughly, thoughtfully, and expertly covered in great detail by others, and we refer readers there for those discussions [75] (and see additional relevant discussion in [63]). Perhaps a fair (albeit oversimplified) consensus that emerged from those discussions might be summarized as follows: While it is reasonable and desirable to devote attention to groups that, for one reason or another, may be more impacted by depression or suicide (or other illness) in order to inform and guide public health efforts aimed at reducing suicide and other consequences of depression, we must also keep an eye on the larger goal, which is broad-scale maximization of individual and public health by minimizing depression and suicide rates and maximizing quality of life for all groups, regardless of race, ethnicity, class, sex, or any other stratifying variable. In other words, we must strive for health equity.

### How do we achieve health equity?

#### Reducing the access gap

One certainty is that we cannot help those we do not reach, and adequate mental health treatment is not reaching many of the subgroups most in need. Thus addressing both the “access gap” and "quality gap" is instrumental to reducing suicide deaths and improving mental health outcomes [1]. Moreover, because health and related inequities and disparities often start early in life and follow one forward, often along racial, ethnic, and/or socioeconomic lines, we must utilize both early childhood prevention efforts and strive to reduce the demonstrated disparities that frequently arise in the mental healthcare of children and young adults [90]. Toward this goal, an excellent and detailed CDC technical package on suicide prevention comments on the high suicide rates among the NHW and AIAN populations [57], and together with others' work [91], recommends a comprehensive and integrated range of multi-factorial approaches for identifying those in need and strengthening access to mental health care across all groups, with the goal of reducing depression and suicide rates in the US population [57, 91].

In addition, with the US’ rising need for mental health treatment, we must address the pronounced shortage of mental health professionals. In the US, 27%, 35%, and 80% of metropolitan, micropolitan, and non-core (i.e., most rural) counties, respectively, lack a psychiatrist [92]. Similar shortage trends exist for psychologists and psychiatric nurse practitioners. Non-core counties consistently have the lowest number of mental health professionals per capita. Across all geographic areas, metropolitan counties (on average) in only five of the nine (5/9) geographic census regions met benchmarks for the ideal number of behavioral health providers needed per 100,000 adults. Only 1/9 census regions met provider benchmarks for micropolitan counties, and 0/9 for non-core counties [92]. Lower-income or poverty are risk factors for both increased depression *and* reduced access to or utilization of care, which represents a complex paradox for delivering effective care in both rural and urban environments. Additionally, although number of providers per capita increases with urbanicity, there are likely to be other risk factors for mental health and suicide and/or contributors to the access gap that are more common to either the urban or rural environments, such as socio-cultural factors and differential access to firearms. These areas require better understanding and improved solutions that can be tailored both individually and by geography.

Another component of this “access gap” and a putative contributing factor to the rising US suicide rates since 1999 is the significant 37% reduction in psychiatric beds from 35 beds per 100,000 population in 1999 to 22 beds per 100,000 in 2013 [93, 94]. Indeed only 4 out of 35 Organization for Economic Cooperation and Development countries (Italy, Chile, Mexico, and Turkey) have fewer psychiatry beds than the US. Psychiatric inpatient care is vital for reducing suicide risk during acute social crises and acute relapse for severe mental illness. The increasingly limited access to acute inpatient care in the US may have resulted in acutely unwell mental health patients being denied essential and potentially life-saving acute treatment, thus putting them at greater risk for suicidal behavior.

Access to outpatient mental health care is equally important, since “the overwhelming majority of mental health services are delivered in outpatient settings” [95]. A recent retrospective study found that a 14% decrease in US community mental health centers from 2014–2017 may be associated with approximately 6% of the increase in US suicide deaths over that same time period [95].

Because of these issues, improving both the quantity and quality of community mental health services is critically important. Another study examined the impact of implementing key mental health service recommendations on suicide rates over time in England and Wales [96]. The average number of recommendations implemented increased from 0.3 to 7.2 per service area from 1998 to 2006. Only the service areas that adopted recommendations had lower suicide rates, and the largest declines in suicide rates were in service areas with the most deprived and largest patient populations [96].

#### Reducing the quality gap

Mental health care, once accessed, must be effective, and current evidence indicates that the “quality gap” [1] encountered in such care may sometimes be considerable. In a review of 1990 to 2015 data from Australia, Canada, England, and the US, Jorm and colleagues found “that the prevalence of mood and anxiety disorders and symptoms has not decreased [in these countries], despite substantial increases in the provision of treatment, particularly antidepressants” [1]. Furthermore, they evaluated several hypotheses for this apparent lack of improvement. They found more support for their hypothesis “that much of the [mental health] treatment provided does not meet the minimal standards of clinical practice guidelines and is not targeted optimally to those in greatest need,” a two-component problem that they termed the “quality gap” [1]. This “quality gap” must be overcome in order to reduce prevalence of common mental health disorders. However they emphasize, and we reiterate, that: “In pointing out that there have not been population mental health gains, we are not suggesting that pharmacological and psychological treatments for common mental disorders do not work. There is abundant evidence from systematic reviews of randomized controlled trials that they do. Rather, this review indicates that there may have been problems of implementation or other factors that may have counteracted their impact” [1].

Indeed, a comprehensive systematic review of numerous studies evidences the apparent efficacies of both antidepressants (as well as a few other drugs) and various psychotherapeutic approaches for suicide prevention [97]. However, some antidepressants may be counter-indicated in select circumstances or populations, especially children and adolescents, and like any drug or medical treatment, are not entirely without risks. Nonetheless, the evidence demonstrates that when appropriately prescribed and managed, antidepressants seem likely to do more good than harm for suicide prevention, particularly in adults and especially in combination with appropriate cognitive therapy [98, 99]. Additional data demonstrating that “suicide rates have tended to decrease more in European countries where there has been a greater increase in the use of antidepressants” also strongly supports this same conclusion [100]. Thus both pharmacologic and psychotherapeutic approaches are crucial and potentially life-saving tools for suicide prevention, particularly when individually tailored and optimally applied. The US must close the access and quality gaps by ensuring those most at risk are identified and receive adequate mental health treatment. To close these gaps, we must do a better job of identifying and mitigating the barriers and other factors that are resulting in both the “access gaps” (i.e., delivery of care to those who need it) and the “quality (or treatment) gaps” (i.e., quality or efficacy of care delivered), and thus preventing optimal mental health outcomes.

#### Precision public health for suicide prevention

Jorm et al. also propose a “prevention gap” resulting from too few resources and efforts devoted to reducing the incidence of mental health disorders through prevention [1]. Indeed, estimates suggest that 20% of US children have a diagnosable mental health condition, and "[m]ore than half of mental illness emerges before age 14" [101], yet 85% of those needing treatment do not receive it [90, 101]. Suicide is the second leading cause of death for 10–34 yo, the fourth leading cause of death for 35–54 yo, and 8th leading cause for ages 55–64 [102]. Put another way, since it is difficult to identify or reach all who may need mental health care (i.e., the access gap), treatments may sometimes be ineffective (the quality gap) or refused, and only 30–48% of suicides (depending on the racial/ethnic group) involve a previously identified mental health condition [74], then it becomes imperative that preventative measures are considered equal first-line defenses for reducing suicide deaths. Much work remains to be done in this critical area.

What can be done to reduce these distressing trends, to improve individual and public health and achieve health equity? We suggest that increased research and understanding of inter- and intra-populational suicide factors are needed in at least three key areas: 1) The underlying contributory (e.g., social, economic, cultural, medical, or other) factors that are driving these trends, 2) Which predisposing factors interact to increase risk and which protective factors reduce risk in the context of predisposing factors – e.g., social, cultural, or biological, and 3) What methods and strategies are most effective for suicide prevention? Some progress has been made in all of these areas, but clearly, much work remains to be done. In this burgeoning era of personalized medicine, a key goal of these efforts should be more effective, individually and community tailored treatment and intervention approaches for preventing suicide deaths, which we believe can only arise from a more complete and integrated understanding of all the factors above. Excellent reviews on suicide’s causes, multi-factorial nature, and limitations of the literature have also recently been published in scientific journals [103] and media [104]. Inherent in finding the answers to these questions is a pressing need for substantially increased funding for suicide prevention research and suicide prevention efforts.

### Is access to firearms a health equity issue?

This section will highlight how access to firearms is associated with higher suicide risk and rates, with noted disparities in both firearm access and suicide based on geography and other factors. Increased or avoidable access to highly lethal means might reasonably be viewed as an environmental determinant to health that is associated with increased suicide risk, thus creating health disparities or "avoidable inequalities" [4] that promote health inequity. Indeed, a health disparity is “a particular type of health difference that is closely linked with social, economic, and/or environmental disadvantage” [4, 105], and access to firearms certainly confers a distinct “environmental disadvantage” when it comes to suicide risk. The abundance of evidence strongly suggests that reducing access to lethal means, particularly firearms, will reduce suicide deaths and save lives, thus advancing health equity, i.e., “attainment of the highest level of health for all people” [4, 105].

#### The “prevention gap” and access to lethal means

The preponderance of studies now find “strong evidence that restricting access to lethal means is associated with a decrease in suicide and that substitution to other methods appears to be limited, which is a major strategy to be integrated into national suicide prevention plans” (quotation from [97]; also see [106]). Moreover, unfortunately yet undeniably, firearms are the most employed lethal means in US suicides, accounting for over half of US suicide deaths in any given year, more than all other lethal means combined [8]. By simple math, this means that strategically and effectively employed firearm safety measures are, by themselves, the preventative actions most likely to have the greatest impact on reducing suicide deaths.

#### Access to firearms and suicide rates

**Fig. 3:**
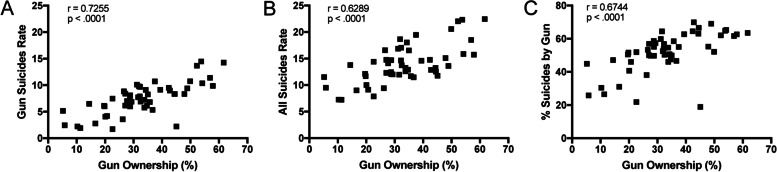
State Gun Ownership versus Gun Suicides, All Suicides, and Percent Suicides by Gun. Spearman correlation analysis for nonparametric data was performed on state gun ownership rates versus state **A** Gun suicide rate, **B** All suicide rate, and **C** Percent suicides by gun. All data was from Table 4. *P* < .0001 for all spearman correlation coefficients (r) shown

Of the 47,511 suicides in the United States in 2019, 23,941 (50%) were by firearm [3]. Easy, immediate access to firearms in the home places household members at 3–5 fold increased risk for suicide [107–109], up to 9-fold increased risk with unsafe storage practices [107], and at 17-fold increased risk for suicide by firearm [108]. Further, this “heightened risk of suicide [with household firearm ownership] is not explained by a higher risk of psychopathology among gun-owning families” [110] (and see [111]). Moreover, “persons with a gun in the home were [are] more likely than others to use a gun to [die by] suicide and less likely than others to [die by] suicide by means of drug overdose, hanging, or other method other than a gun” [108]. Population (household) rates of gun ownership are strongly associated with overall suicide rates, gun suicide rates, and percentage of suicides by gun in the US (Table 4 and Fig. 3) [112–114], even when prescription rates of antidepressants are considered [115]. Evidence suggests that 33%-80% of suicidal acts are impulsive [116]. Among individuals making near-lethal suicide attempts, 70% made the decision within an hour of the attempt, and 24% within 5 min of the attempt [117]. Furthermore, “more than 90% of people who survive a suicide attempt, including attempts that were expected to be lethal... do not go on to die by suicide” [116] – thus strongly supporting the emerging understandings that suicide is not inevitable, and that only very rarely will suicide attempters substitute another lethal means if one is not readily available [106, 118].

**Table 4:** State Gun Ownership versus Gun Suicides, All Suicides, and Percent Suicides by Gun

State	%Gun Owners	GO Rank	Gun Suicides	GS Rank	All Suicides	AS Rank	%Gun Suicides	%GS Rank
Alaska	61.7	1	14.26	2	22.45	1	63.5	8
Arkansas	57.9	2	9.86	9	15.74	14	62.6	11
Idaho	56.9	3	11.39	4	18.51	7	61.6	13
West Virginia	54.2	4	10.36	7	15.87	13	65.3	4
Wyoming	53.8	5	14.47	1	22.33	2	64.8	5
Montana	52.3	6	13.67	3	22.04	3	62.0	12
New Mexico	49.9	7	10.73	5	20.58	4	52.1	27
Alabama	48.9	8	9.40	13	13.62	25	69.0	2
North Dakota	47.9	9	8.34	20	15.10	15	55.2	20
Hawaii	45.1	10	2.22	47	11.76	38	18.9	50
Louisiana	44.5	11	8.35	19	12.54	31	66.6	3
South Carolina	44.4	12	8.34	21	13.24	27	63.0	9
Mississippi	42.8	13	8.99	16	12.86	29	69.9	1
Kentucky	42.4	14	9.52	12	14.76	17	64.5	6
Tennessee	39.4	15	9.14	14	14.59	20	62.7	10
Nevada	37.5	16	10.71	6	19.46	5	55.0	22
Minnesota	36.7	17	5.34	40	11.46	42	46.6	39
Texas	35.7	18	6.82	32	11.67	40	58.5	16
South Dakota	35	19	8.25	22	16.53	12	49.9	34
Wisconsin	34.7	20	6.13	35	12.90	28	47.5	37
Colorado	34.3	21	9.12	15	18.04	8	50.6	30
Indiana	33.8	22	7.23	28	13.25	26	54.6	23
Iowa	33.8	23	5.79	39	12.59	30	46.0	41
Florida	32.5	24	6.90	30	13.73	24	50.3	32
Arizona	32.3	25	9.68	11	17.05	9	56.8	19
Kansas	32.2	26	7.88	24	14.64	19	53.8	24
Utah	31.9	27	9.79	10	18.66	6	52.5	26
Georgia	31.6	28	7.71	25	11.96	37	64.4	7
Oklahoma	31.2	29	10.09	8	16.86	10	59.8	14
Virginia	29.3	30	6.89	31	12.02	36	57.3	17
Vermont	28.8	31	8.10	23	14.69	18	55.1	21
Michigan	28.8	32	5.99	38	12.05	35	49.7	36
North Carolina	28.7	33	7.28	27	12.42	32	58.6	15
Washington	27.7	34	7.02	29	14.11	22	49.7	35
Missouri	27.1	35	8.41	18	14.77	16	56.9	18
Pennsylvania	27.1	36	6.15	34	12.30	33	50.0	33
Oregon	26.6	37	8.84	17	16.57	11	53.3	25
Illinois	26.2	38	3.58	44	9.40	45	38.1	44
Maine	22.6	39	7.47	26	14.40	21	51.9	28
Massachusetts	22.6	40	1.71	50	7.86	48	21.8	49
Maryland	20.7	41	4.20	42	9.13	46	46.0	40
California	20.1	42	4.07	43	10.00	43	40.7	43
Nebraska	19.8	43	6.02	37	11.67	39	51.6	29
Ohio	19.6	44	6.12	36	12.11	34	50.5	31
Connecticut	16.6	45	2.79	45	9.01	47	31.0	45
New Hampshire	14.4	46	6.49	33	13.78	23	47.1	38
New Jersey	11.3	47	1.92	49	7.24	50	26.5	47
New York	10.3	48	2.21	48	7.26	49	30.4	46
Rhode Island	5.8	49	2.45	46	9.52	44	25.8	48
Delaware	5.2	50	5.16	41	11.50	41	44.9	42

All data is age-adjusted deaths per 100,000 (2000 US population) and cumulative 1999–2019 data [3], except survey-based gun ownership by state [119], and percent gun suicides (= gun suicide rate/all suicide rate)

Finally, a recent report in the New England Journal of Medicine is unique in its large size, its control of potentially confounding variables, and its analysis of the impact of first-time personal handgun ownership on an individual’s risk of dying by suicide over time. This seminal study found that compared to nonowners, handgun owners had 3.67 times higher suicide rates (3.34 × for males, 7.16 × for females), and 9.08 times higher firearm suicide rates (7.82 × males, 35.15 × females) (adjusted hazard ratios) [120]. This significantly increased risk of suicide in first-time handgun owners was driven by much higher rates of firearm suicide (not by higher rates of suicide by other means), which peaked in the period immediately after gun acquisition and remained elevated after 12 years (the duration of the study). The authors concluded: "Our study bolsters and extends the message from previous research: ready access to firearms, particularly handguns, is a major risk factor for suicide" [120].

Collectively, the data convincingly demonstrate that 1) aggregate and individual suicide risks are at least three times higher with firearm access than without, 2) these increased risks of suicide with firearm access are driven by higher rates of firearm suicides, not higher rates of suicide by other means, and 3) means substitution is rare and most likely insufficient to keep overall suicide rates constant if firearm suicide rates were to decline. As has been aptly summarized regarding suicide attempts: "about 85% of people who use a gun will die; about 95% of people who use another means will survive; and more than 90% of those who survive will not go on to try again" [121]. In short, a more cautious approach to firearm access would be highly likely to reduce overall suicide deaths in the US.

Consistent with this data, firearm suicide rates also appear to track household gun ownership rates across racial/ethnic groups. In one study, Riddell et al. explored the relationships between rates of firearm suicide and reported household firearm ownership in NHW and NHB, by state, and concluded that “state gun prevalence was strongly tied to firearm suicide for both black and white men” [122]. Furthermore, of US adults surveyed in April 2017, 49% (NHW), 32% (NHB), and 21% (HISP) reported the presence of a gun in their household [123]. API consistently have the lowest reported household gun ownership, estimated at 15% in one survey [124]. In contrast, little household gun ownership data is available for AIAN but was reported at 53% in a small sample size (148 respondents) from two localities in Alaska, slightly under the percentage of NHW for the regions sampled [125]. Similarly, firearm suicide rates (both sexes, all ages, per 100,000 age-adjusted population) for these groups in 2017 follow the same rank order: 9.1 (NHW), 8.4 (AIAN), 3.4 (NHB), 2.5 (HISP), and 1.7 (API). Together with Tables 2–3 and Fig. 3, these data suggest that gun (and overall) suicide rates appear to closely track gun ownership rates across different populations.

Geographic differences in firearm ownership, both internationally and nationally in the US, are another likely factor in the refractory US suicide rate. US gun ownership is very high by international standards, and the overall US rate of firearm suicide (6.9 per 100,000 age-adjusted population in 2017; 6.1 in 2010 for comparison below) [8] is far higher than any other high-income countries [126], and far higher than rates in countries with more robust firearm safety measures such as the United Kingdom (0.2 per 100,000), Australia (0.8), New Zealand (1.0), and Canada (1.7) [126]. Similarly, firearm ownership, suicide rates, and firearm suicide rates are all considerably lower on average in Europe versus the US, and European countries with higher gun ownership rates also tend to have higher gun suicide rates than European countries with lower gun ownership rates [127].

Here in the US, suicide rates rise significantly with increasing rurality (i.e., lowest in cities, higher in towns/small cities, highest in rural areas) (Fig. 2, Tables 1–3) [8], where gun ownership rates are higher (Table 4). Accordingly, the rural states consistently have the highest rates of gun ownership, total suicides, and firearm suicides in the US, whereas the states with the lowest household gun ownership (and usually more protective firearm laws), which are often more densely populated, tend to have lower suicide rates (Table 4) [128–131]. Indeed, for each state, survey-based estimates of gun ownership (% individuals) are strongly and significantly correlated with gun suicide rate, overall suicide rate, and percent suicides by gun within the state (Fig. 3). This is consistent with others' validation of percent suicides by gun as a proxy measure for household firearm ownership prevalence [132]. Although in our analysis using survey-based data for state gun ownership rates [119] together with cumulative (1999–2019) age-adjusted data for statewide suicide and firearm suicide rates [3], we found the strongest correlation between gun ownership and gun suicide rates (*r* = 0.7255, *p* < 0.0001), followed by % suicides by guns (*r* = 0.6744, *p* < 0.0001), and overall suicides (*r* = 0.6289, *p* < 0.0001).

Males, those living in rural states or areas, and select other populations such as AIAN and NHW, all have higher suicide and gun suicide rates – and typically higher firearm ownership – versus other groups (Fig. 2, Tables 1–3). Older men also have disproportionately high gun suicide rates and percent suicides by gun, and NHB males match NHW males in having the highest percent suicides by gun (despite NHB males having relatively lower overall suicide rates) (Table 2). Rural males making a living in agriculture may also be at particular risk [133, 134]. For these reasons alone, some groups may see more relative benefit from firearm safety measures, but all groups will benefit from such measures by reduced suicide deaths. Also, while firearms typically account for ≥ 50% of suicides across both sexes, females die by suicide at a lower rate than males, and less frequently with firearms than males (typically < 50% of female suicides are by firearm, versus > 50% for males) (Table 2) [135]. Therefore, additional strategies to reduce access to other lethal means (besides firearms) will also help minimize suicide and maximize public health for all groups, but with a relatively more significant benefit for women.

Nonetheless, a recent seminal study showed that personal handgun ownership increased women's individual risks for suicide and firearm suicide even more so than for men (compared to female and male nonowners, respectively) [120]. From this, the authors posit that "handgun ownership may impose a particularly high relative risk of suicide for women because of the pairing of their higher propensity to attempt with ready access to and familiarity with an extremely lethal method" [120]. From these data, one might also hypothesize that part of the reason men have higher suicide and firearm suicide rates versus women may be that, on average, men have greater exposure to and familiarity with (and less aversion to?) firearms than women. In other words, when it comes to suicide risk, easy and familiar access (i.e., desensitization) to firearms for women, where it exists, may be an even more dangerous proposition than for men.

#### Identifying the most effective firearm safety measures

We approach this topic from purely a public health perspective. Based upon the preponderance of evidence, we wish to emphasize the point, as others have, that reducing access to lethal means will almost certainly save many lives that might otherwise be lost to suicide [97, 113, 114, 118, 120, 131, 136]. Especially because since 1999, suicides have accounted for 55%-64% of all US firearm deaths on average, with that number being ≥ 60% since 2010 [137, 138]. In many states, 80–90% of gun deaths are suicides [139].

Reducing access to lethal means as pertains to firearms in particular can take many forms, ranging from individual to community to legislative initiatives. Universal background checks (UBCs) limit firearm purchases to only individuals of qualified age, and without other risk factors such as substance abuse, threats to self or others due to mental illness, or convictions of crimes of violence or domestic violence. Anestis & Anestis (2015) found states that utilize UBCs had about a 50% lower rate of firearm suicides [140]. By limiting easy/immediate acquisition of firearms, other similar regulations that delay or deter gun purchases such as mandatory waiting periods or permit requirements, are also linked to reductions in suicide deaths. Specifically, waiting periods "lead to a 7–11% reduction in gun suicides... which is equivalent to 22–35 fewer gun suicides per year for the average state" [141].

Permit to purchase (PTP) laws are similar to UBC laws as they typically require a background check to obtain a permit, and have been associated with a 15% reduction in firearm suicide rate [142]. Further, this study by Crifasi et al. [142] estimated the effects of changes in PTP handgun laws in Connecticut and Missouri. Connecticut adopted a permit-to-purchase handgun law in October, 1995 that included mandated background PTP for all handgun transfers, contingent on passing a background check and safety training course. Missouri had a PTP handgun law since the 1920s that required a PTP for all handgun sales, with permits obtained from the sheriff and good for 30 days. This law was repealed in Aug. 28, 2007. In Connecticut, firearm suicide rates declined by 15% in the decade after the PTP law was introduced. In stark contrast, firearm suicide rates rose by 16% in the five years after the PTP law was repealed in Missouri [142].

The CDC’s technical package on suicide prevention recommends safe (i.e., locked) storage practices for firearms and other lethal means (e.g., drugs and toxins) as preventative measures for at-risk individuals, and provides evidence that such safe storage practices have been found effective for reducing suicide risk [57]. In support, others have presented comprehensive meta-analyses of approaches to reducing access to various lethal means that have been demonstrably effective for reducing suicide risk [97]. Regarding firearms in particular, significant evidence indicates that safe storage helps reduce suicide risk [107, 113, 143], with suicide risk increasing predictably with each gradation of unsafe storage. For example, versus “no guns in the home,” having “any gun kept unlocked” or “any gun kept loaded” increased all-means suicide risk by 5.6-fold and 9.2-fold, respectively [107]. Others found that adolescent suicide was four times more likely in homes with unlocked and loaded firearms, than in homes with locked and unloaded firearms [144, 145]. Policies that focus on responsible firearm storage, such as Child Access Prevention laws, are associated with a 19% decrease in gun suicides among ages 10–14 and an 11% decrease among ages 14–17 [146–148].

In part for these reasons, firearm personalization or “smart gun” technology, which limits firearm use to the owner or permitted user, is a promising and evolving technology with the potential to reduce suicide deaths, perhaps especially youth firearm suicides [114]. Several excellent overviews of the range of firearm safety measures that have been shown to reduce suicide risk are available [97, 113, 114].

Thus, where firearms are present, maximizing the use of both safe storage practices (i.e., locked, unloaded, and with ammunition stored separately) and all other available firearm safety measures is critical, and can significantly reduce suicide risk compared to less safe firearm practices. At the same time, perfect or “ideal” safe-storage compliance and gun safety in all households with firearms is both difficult to achieve and subject to opinion [149, 150], and even with “all guns locked up,” suicide risk is still 2.4 times higher than in households without firearms [107]. Hence supplementary legislative firearm safety measures may further help minimize suicide deaths and maximize public health and health equity.

Extreme risk protection orders (ERPOs), lethal violence protection orders (LVPOs), and gun violence restraining orders (GVROs) temporarily prohibit access to firearms (purchase or possession) by high-risk individuals. These programs enable law enforcement, health professionals, and families to intervene when individuals are dangerous to themselves or others. ERPOs or GVROs were associated with a 7.5% reduction of firearm suicide rate in Indiana and a 14% reduction in Connecticut [151].

Clinicians can also provide lethal means counseling to high-risk patients and their family or friends support systems, to limit access until they are no longer at elevated risk [152]. One such program, Counseling on Access to Lethal Means, trained emergency department providers to counsel families of youths at high risk for suicide on restricting home access to lethal medications, alcohol, and firearms. In this program, 76% of parents contacted at follow-up reported all medications in the home were locked as compared to fewer than 10% at the time of the first visit, and 100% indicated guns in the home were currently locked, as compared to 67% reporting this at the time of the initial visit [153]. These programs have great potential to reduce youth and adult suicide rates through educational programs regarding safe storage practices of potentially lethal means, particularly around individuals at high risk of self-harm.

Awareness [154] or legislative [142] campaigns to help prevent firearms sales to at-risk individuals may also be helpful. Directly engaging key stakeholders in gun shop prevention programs have a track record of success and hold much promise [121, 155]. Those programs provide suicide prevention literature within the store, and educate gun shop owners on spotting suicide risks and then taking safe and appropriate preventive action (e.g., refusing a sale, talking with the customer and referring them to suicide prevention resources if appropriate). An increased presence of gun shops is associated with higher suicide rates, underscoring the need for more lethal means safety programs of this nature [79]. These are all very positive steps in the right direction with demonstrated efficacy in reducing suicide deaths [142, 151, 156]; thus, the importance of these measures for improving public health cannot be overstated.

Perhaps not surprisingly then, by some reports, "empirical analysis suggest[s] that firearms regulations which function to reduce overall gun availability have a significant deterrent effect on male suicide, while regulations that seek to prohibit high risk individuals from owning firearms have a lesser effect” [157]. So in some cases, additional proactive safety measures to reduce access to lethal means may be necessary to truly minimize suicide deaths and maximize public health. Many rigorous analyses suggest that these kinds of additional safety measures are effective for reducing suicides, saving lives, and promoting health equity [113, 114, 118, 131].

Also encouraging is the recent legislative guidance or “clarification” that the Dickey Amendment does not prevent the use of federal funds for research on gun violence. A careful reading of the Dickey Amendment would indeed agree that, strictly speaking, it never did. Instead, it simply stated “That none of the funds made available for injury prevention and control at the Centers for Disease Control and Prevention may be used to advocate or promote gun control” – i.e., it only prohibited firearm control advocacy with those federal CDC funds [158]. However, the common misconception that the Dickey Amendment prohibited or “banned” the use of federal funds for gun violence research probably did not arise by accident. Rather, passage of the Amendment in 1996 was accompanied by a $2.6 million reduction in the CDC’s budget, equivalent to the exact amount the CDC had spent on firearm-related research the previous year [159]. The message seemed clear and effectively squelched any subsequent federally funded research on gun violence, including nearly all extramural National Institutes of Health (NIH)-funded research on gun violence despite the massive public health problem that it represents, putatively because no federal agency has been willing to risk the potential political or budgetary repercussions should they fund or participate in such research [160, 161]. (The sparse NIH funds that have gone toward investigating gun violence over the years are a positive step forward [162], but still a drop in the bucket compared to what the NIH spends on equivalent public health threats [163–165].) Therefore, although the recent legislative “clarification” on what is permitted under the Dickey Amendment is a welcome step in the right direction, in effect nothing has changed, and it remains to be seen whether this recent clarifying guidance will translate to increased availability and distribution of federal funds for research on firearm suicide and other gun violence. Encouragingly, there have been some small but important steps in the right direction, and the majority of people want to get on board with improving safety, reducing deaths, and maximizing individual and public health [165, 166].

### Suicide and COVID-19

On March 11, 2020, the “Coronavirus Disease 2019” (“COVID-19”) outbreak was declared a pandemic by the World Health Organization [167]. Mass isolation orders were implemented in states without a concrete understanding of how long those mitigation measures would be required to adequately impact mortality and morbidity. For many Americans during that time, their context was characterized by social isolation from physical distancing, more limited access to basic needs, anxiety, uncertainty about contracting the virus and infecting others, bereavement, job loss, interrupted education, and sensationalized and anxiety-provoking media messages.

Key population risk factors for suicide include a lack of social cohesion, such as rapid changes in social structure, economic turmoil, and social isolation [168], which many Americans have experienced in the context of COVID-19. Other key environmental risk factors for suicide are ready access to lethal means, and poor access to mental health care. A record high 21 million background checks for firearm sales were conducted in 2020, with 40% of those for first-time gun owners [169–171]. Simultaneously, access to mental health care has been reduced [172]. Together these factors may result in the onset or worsening of anxiety, depression, and post-traumatic stress disorders, and increased suicide risk, among the general population, health care professionals, and other front-line service workers during COVID-19 [173, 174].

Some early and mixed reports are emerging. During the COVID pandemic to date, overall suicides in many regions have remained steady or declined in both the US [58, 175, 176] and worldwide [177], despite evidence for increased suicide risk factors and mental health distress during this period [177, 178]. However, the prolonged stressors associated with the pandemic could result in increased suicides among certain subgroups most negatively impacted, and/or delayed impacts on overall suicide rates [179]. For example, from early March to early May of 2020 in Maryland, suicides increased by 94% amongst NHB while declining 45% among NHW, compared to the same time period averaged over the prior three years [180, 181]. Perhaps tellingly, gun sales to African Americans increased 58% in 2020 versus 2019, the largest increase for any demographic category [170]. In the summer of 2020 (July 26–August 22, 2020) and winter of 2021 (February 21–March 20, 2021), suicide attempts by US adolescents aged 12–17 yo, particularly girls, spiked as much as 51% compared to the equivalent prior year time period [182]. Other countries also saw similar increases in adolescent suicide attempts during the COVID-19 pandemic year, compared to the prior year. From March 2020 to March 2021, adolescent suicide attempts in Spain increased 25%, versus a 16.5% decline for adult suicide attempts [183]. As with the US data, Spanish adolescent girls experienced the greatest increase in suicide attempt rates, which increased as much as 195% from September 2020 – March 2021, compared to the prior year [183]. Lower income countries may be more susceptible to increased suicide rates associated with the COVID-19 pandemic [177]. Readers may refer to [184] for very comprehensive additional discussions of the impact of COVID-19 on worldwide suicide. Collectively, these data suggest potential health disparities in COVID-19’s impact on suicide risk, and warrant further investigation as data becomes available.

Unfortunately, there is already evidence emerging that Anti-Asian racism and hate crimes have increased as a result of COVID-19 [185]. There appears to be a widening of the health disparity gap in terms of COVID-19 morbidity and mortality. COVID-19 has had a disproportionally negative impact on morbidity and mortality in African Americans, Latinx individuals, and Native Americans [186]. Access to some COVID-19 public health testing is available only by drive-through options, exacerbating disparities in accessing needed care. Many school systems have struggled to provide equitable and inclusive online education to those without WIFI access and devices that connect to the internet.

After acute turmoil, the impact of increased suicide is often not seen immediately. Based on the Severe Acute Respiratory Syndrome (SARS) experience in Hong Kong, suicide rates increased among older adults by 30% following the SARS outbreak [187, 188]. An increase in suicide could become a concern either during or post-pandemic, as the pandemic’s longer-term impacts on the general population, economy, and vulnerable groups become more clear [173]. Healthcare workers presently caring for, or that have cared for, COVID-19 patients may be at particular risk for suicide, trauma, post-traumatic stress disorder, anxiety, depression, and other acute or chronic impacts on their mental health [189, 190]. The pandemic’s suicide-related consequences may vary depending on local and national public health control measures implemented, including the availability of evidence-based telemedicine and digital alternatives to face-to-face mental health care at scale, and existing outreach with clear pathways to care for those at risk and other supports [173]. To achieve health equity, we must investigate and understand the differing risk equations for different populations, and ensure that the necessary resources are in place to mitigate those risks for the benefit of individual and public health.

## Conclusions

Significant and often underappreciated disparities in suicide deaths occur among disproportionately at-risk populations including children and young adults, the elderly, veterans, AIAN and NHW, inhabitants of rural areas, and those with increased access to firearms. Our analysis of the literature and underlying factors suggests that many of these deaths are likely to be preventable by taking a public health approach that includes: 1) implementing and sustaining evidence-based upstream prevention strategies that start long before someone becomes suicidal, 2) enhancing access to effective treatments for depression, and 3) common-sense lethal means reductions for those most at risk. A recent report by US Congress Joint Economic Committee staff, entitled "Guns and Suicide," has highlighted findings and drawn conclusions similar to ours herein [191], providing hope that these issues may finally receive greater and much needed legislative attention. There are a few examples of European countries that have achieved sustained reductions in their suicide rates by taking these types of population-level approaches. England, Finland, and Denmark have invested resources to carefully address suicide at the population level, by using multiple coordinated elements to reduce access to lethal means while enhancing access to mental health services [192–194]. Furthermore, sensible and situationally/geographically appropriate means reductions approaches are demonstrably effective for reducing suicides by highly lethal means [97, 106, 114, 118, 130, 131, 142, 143, 151, 156, 157, 195]. Yet similar large-scale, integrated, and coordinated public health approaches to suicide prevention has not yet been widely applied in the United States. We hope that increased national prioritization of all these critical areas can help stem the rapidly rising tide of US suicides and improve health equity.

## Data Availability

All data generated or analysed during this study are included and/or referenced in this published article.

## Abbreviations

AIAN: American Indian/Alaska Native
API: Asian/Pacific Islander
CDC: Centers for Disease Control and Prevention
COVID-19: Coronavirus Disease 2019
ERPOs: Extreme risk protection orders
GVROs: Gun violence restraining orders
HISP: Hispanic
LVPOs: Lethal violence protection
Metro: Metropolitan
NHB: Non-Hispanic black
NHW: Non-Hispanic white
NIH: National Institutes of Health
NSDUH: National Survey on Drug Use and Health
PTP: Permit to purchase
PYMD: Past-year major depressive episode
SARS: Severe Acute Respiratory Syndrome
SMR: Standardized mortality ratio
SSRI: Selective serotonin reuptake inhibitor
UBCs: Universal background checks
VA: US Department of Veterans Affairs

## References

[1] Jorm AF, Patten SB, Brugha TS, Mojtabai R (2017). Has increased provision of treatment reduced the prevalence of common mental disorders? Review of the evidence from four countries. World Psychiatry.

[2] Perry SW, Allison S, Bastiampillai T, Wong M-L, Licinio J, Sharfstein SS, Wilcox HC: Rising US Suicides: Achieving Health Equity. OSF Preprints, November 22, 2019 1031219/osfio/m5q64

[3] National Center for Health Statistics, Mortality Data on CDC WONDER [https://www.wonder.cdc.gov/ucd-icd10.html]

[4] National Stakeholder Strategy for Achieving Health Equity. Rockville, MD: By: National Partnership for Action to End Health Disparities, US Department of Health and Human Services, Office of Minority Health; 2011 (https://www.minorityhealth.hhs.gov/npa/files/Plans/NSS/CompleteNSS.pdf).

[5] Hedegaard H, Curtin SC, Warner M (2018). Suicide Mortality in the United States, 1999–2017. NCHS Data Brief.

[6] Hedegaard H, Curtin SC, Warner M (2020). Increase in suicide mortality in the United States, 1999–2018. NCHS Data Brief.

[7] Suicide mortality in the United States, 1999–2019. In. Edited by National Center for Health S. Hyattsville, MD: http://dx.doi.org/10.15620/cdc:101761; 2021.

[8] Ivey-Stephenson AZ, Crosby AE, Jack SPD, Haileyesus T, Kresnow-Sedacca MJ (2017). Suicide trends among and within urbanization levels by sex, race/ethnicity, age group, and mechanism of death - United States, 2001–2015. MMWR Surveill Summ.

[9] CDC Fact Sheet: Health Disparities in Suicides. Atlanta, GA: By: Centers for Disease Control and Prevention (CDC), US Department of Health and Human Services; 2011 (https://www.cdc.gov/minorityhealth/chdir/2011/factsheets/suicide.pdf).

[10] Rockett IRH, Caine ED, Connery HS, Nolte KB, Nestadt PS, Nelson LS, Jia H (2020). Unrecognised self-injury mortality (SIM) trends among racial/ethnic minorities and women in the USA. Inj Prev.

[11] Rockett IRH, Caine ED, Connery HS, D'Onofrio G, Gunnell DJ, Miller TR, Nolte KB, Kaplan MS, Kapusta ND, Lilly CL (2018). Discerning suicide in drug intoxication deaths: Paucity and primacy of suicide notes and psychiatric history. PLoS ONE.

[12] Ali B, Rockett IRH, Miller TR, Leonardo JB (2022). Racial/ethnic differences in preceding circumstances of suicide and potential suicide misclassification among US adolescents. J Racial Ethn Health Disparities.

[13] Presentation of the publicly available National Center for Health Statistics Mortality Data from CDC WONDER (https://wonder.cdc.gov/ucd-icd10.html) in this section is mainly descriptive, for illustrative purposes. Any group differences in the level and temporal changes in suicide rates were not formally examined using statistical tests.

[14] Benton TD (2022). suicide and suicidal behaviors among minoritized youth. Child Adolesc Psychiatr Clin N Am.

[15] Karaye IM (2022). Differential trends in US suicide rates, 1999–2020: emerging racial and ethnic disparities. Prev Med.

[16] Ramchand R, Gordon JA, Pearson JL (2021). Trends in suicide rates by race and ethnicity in the United States. JAMA Netw Open.

[17] Xiao Y, Cerel J, Mann JJ (2021). temporal trends in suicidal ideation and attempts among US adolescents by sex and race/ethnicity, 1991–2019. JAMA Netw Open.

[18] Polanco-Roman L: Suicide-Related Risk among Racial and Ethnic Minority Youth: Important Considerations. Blog article, originally published 1/5/2020, accessed 05/17/2022: https://www.youthsuicideresearchorg/blog/suicide-related-risk-among-racial-and-ethnic-minority-youthnbspimportant-considerationsblog/youthresearchorg.

[19] Curtin SC, Heron M: Death rates due to suicide and homicide among persons aged 10–24: United States, 2000–2017. NCHS Data Brief, no 352 Hyattsville, MD: National Center for Health Statistics 2019. 31751202

[20] Bostwick WB, Meyer I, Aranda F, Russell S, Hughes T, Birkett M, Mustanski B (2014). Mental health and suicidality among racially/ethnically diverse sexual minority youths. Am J Public Health.

[21] di Giacomo E, Krausz M, Colmegna F, Aspesi F, Clerici M (2018). Estimating the risk of attempted suicide among sexual minority youths: a systematic review and meta-analysis. JAMA Pediatr.

[22] Hottes TS, Bogaert L, Rhodes AE, Brennan DJ, Gesink D (2016). Lifetime prevalence of suicide attempts among sexual minority adults by study sampling strategies: a systematic review and meta-Analysis. Am J Public Health.

[23] Johns MM, Lowry R, Rasberry CN, Dunville R, Robin L, Pampati S, Stone DM, Mercer Kollar LM (2018). Violence victimization, substance use, and suicide risk among sexual minority high school students - United States, 2015–2017. MMWR Morb Mortal Wkly Rep.

[24] King M, Semlyen J, Tai SS, Killaspy H, Osborn D, Popelyuk D, Nazareth I (2008). A systematic review of mental disorder, suicide, and deliberate self harm in lesbian, gay and bisexual people. BMC Psychiatry.

[25] Marshall A (2016). Suicide prevention interventions for sexual & gender minority youth: an unmet need. Yale J Biol Med.

[26] Mereish EH, Sheskier M, Hawthorne DJ, Goldbach JT (2019). Sexual orientation disparities in mental health and substance use among Black American young people in the USA: effects of cyber and bias-based victimisation. Cult Health Sex..

[27] Puckett JA, Horne SG, Surace F, Carter A, Noffsinger-Frazier N, Shulman J, Detrie P, Ervin A, Mosher C (2017). Predictors of sexual minority youth's reported suicide attempts and mental health. J Homosex.

[28] Shadick R, Backus Dagirmanjian F, Barbot B (2015). Suicide risk among college student. the intersection of sexual orientation and race. Crisis..

[29] Fish JN, Rice CE, Lanza ST, Russell ST 2018 Is young adulthood a critical period for suicidal behavior among sexual minorities? results from a US national sample. Prev Sci 10.1007/s11121-018-0878-5 PMC6163093 29594980

[30] Sexton MB, Davis MT, Anderson RE, Bennett DC, Sparapani E, Porter KE (2018). Relation between sexual and gender minority status and suicide attempts among veterans seeking treatment for military sexual trauma. Psychol Serv.

[31] Blosnich JR, Mays VM, Cochran SD (2014). Suicidality among veterans: implications of sexual minority status. Am J Public Health.

[32] Haas AP, Lane AD, Blosnich JR, Butcher BA, Mortali MG: Collecting Sexual Orientation and Gender Identity Information at Death. Am J Public Health 2018:e1-e5 10.2105/AJPH.2018.304829 PMC6336043 30571294

[33] Pompili M, Lester D, Forte A, Seretti ME, Erbuto D, Lamis DA, Amore M, Girardi P (2014). Bisexuality and suicide: a systematic review of the current literature. J Sex Med.

[34] Salway T, Ross LE, Fehr CP, Burley J, Asadi S, Hawkins B, Tarasoff LA (2019). A systematic review and meta-analysis of disparities in the prevalence of suicide ideation and attempt among bisexual populations. Arch Sex Behav.

[35] Horwitz AG, Berona J, Busby DR, Eisenberg D, Zheng K, Pistorello J, Albucher R, Coryell W, Favorite T, Walloch JC (2020). Variation in suicide risk among subgroups of sexual and gender minority college students. Suicide Life Threat Behav.

[36] 2019 National Veteran Suicide Prevention Annual Report [https://www.mentalhealth.va.gov/docs/data-sheets/2019/2019_National_Veteran_Suicide_Prevention_Annual_Report_508.pdf]

[37] Sall J, Brenner L, Millikan Bell AM, Colston MJ (2019). Assessment and management of patients at risk for suicide: synopsis of the 2019 U.S. department of veterans affairs and U.S. department of defense clinical practice guidelines. Ann Intern Med.

[38] VA/DoD Clinical Practice Guidelines: Assessment and Management of Patients at Risk for Suicide (2019) [https://www.healthquality.va.gov/guidelines/MH/srb/]

[39] Hogan M (2019). Veteran suicide: not just a va issue; it's a US issue. Ann Intern Med..

[40] Department of Veterans Affairs. Suicide among Veterans and other Americans 2001–2014. Washington, DC, Office of Suicide Prevention [https://www.mentalhealth.va.gov/docs/2016suicidedatareport.pdf]

[41] Veteran Outreach Toolkit. Preventing Veteran Suicide Is Everyone’s Business: A Community Call to Action [https://www.va.gov/ve/docs/outreachToolkitPreventingVeteranSuicideIsEveryonesBusiness.pdf]

[42] Speer M, Phillips MA, Winkel T, Wright W, Winkel N, Reddy S: “Serving Those Who Serve: Upstream Intervention And The Uphill Battle Of Veteran Suicide Prevention In The US, " Health Affairs Blog, July 11, 2019 10.1377/hblog20190709.197658

[43] Executive Order on a National Roadmap to Empower Veterans and End Suicide [https://www.whitehouse.gov/presidential-actions/executive-order-national-roadmap-empower-veterans-end-suicide/]

[44] Kegler SR, Stone DM, Holland KM (2017). Trends in suicide by level of urbanization - United States, 1999–2015. MMWR Morb Mortal Wkly Rep.

[45] Rossen LM, Hedegaard H, Khan D, Warner M (2018). County-level trends in suicide rates in the US, 2005–2015. Am J Prev Med..

[46] Fontanella CA, Hiance-Steelesmith DL, Phillips GS, Bridge JA, Lester N, Sweeney HA, Campo JV (2015). Widening rural-urban disparities in youth suicides, United States, 1996–2010. JAMA Pediatr.

[47] Singh GK, Siahpush M (2002). Increasing rural-urban gradients in US suicide mortality, 1970–1997. Am J Public Health.

[48] NCHS Urban-Rural Classification Scheme for Counties [https://www.cdc.gov/nchs/data_access/urban_rural.htm] 22783637

[49] Hoffmann JA, Farrell CA, Monuteaux MC, Fleegler EW, Lee LK (2020). Association of pediatric suicide with county-level poverty in the United States, 2007–2016. JAMA Pediatr.

[50] Naher AF, Rummel-Kluge C, Hegerl U (2019). Associations of suicide rates with socioeconomic status and social isolation: findings from longitudinal register and census data. Front Psychiatry.

[51] Zheng L, Wang O, Hao S, Ye C, Liu M, Xia M, Sabo AN, Markovic L, Stearns F, Kanov L (2020). Development of an early-warning system for high-risk patients for suicide attempt using deep learning and electronic health records. Transl Psychiatry.

[52] Baldessarini RJ (2019). Epidemiology of suicide: recent developments. Epidemiol Psychiatr Sci.

[53] Mann JJ, Metts AV (2017). The economy and suicide. Crisis.

[54] Bachmann S (2018). Epidemiology of suicide and the psychiatric perspective. Int J Environ Res Public Health.

[55] Kerr WC, Kaplan MS, Huguet N, Caetano R, Giesbrecht N, McFarland BH (2017). economic recession, alcohol, and suicide rates: comparative effects of poverty, foreclosure, and job loss. Am J Prev Med.

[56] Purselle DC, Heninger M, Hanzlick R, Garlow SJ (2009). Differential association of socioeconomic status in ethnic and age-defined suicides. Psychiatry Res.

[57] Stone DM, Holland KM, Bartholow B, Crosby AE, Davis S, Wilkins N: Preventing Suicide: A Technical Package of Policies, Programs, and Practices. In., edn. Atlanta, GA: National Center for Injury Prevention and Control, Centers for Disease Control and Prevention; 2017: https://www.cdc.gov/violenceprevention/pdf/suicideTechnicalPackage.pdf.

[58] Ehlman DC, Yard E, Stone DM, Jones CM, Mack KA (2022). Changes in suicide rates - United States, 2019 and 2020. MMWR Morb Mortal Wkly Rep.

[59] Does depression increase the risk for suicide? [https://www.hhs.gov/answers/mental-health-and-substance-abuse/does-depression-increase-risk-of-suicide/index.html]

[60] Too LS, Spittal MJ, Bugeja L, Reifels L, Butterworth P, Pirkis J (2019). The association between mental disorders and suicide: a systematic review and meta-analysis of record linkage studies. J Affect Disord.

[61] Chesney E, Goodwin GM, Fazel S (2014). Risks of all-cause and suicide mortality in mental disorders: a meta-review. World Psychiatry.

[62] Substance Abuse and Mental Health Services Administration. (2020). Key substance use and mental health indicators in the United States: Results from the 2019 National Survey on Drug Use and Health (HHS Publication No. PEP20–07–01–001, NSDUH Series H-55). Rockville, MD: Center for Behavioral Health Statistics and Quality, Substance Abuse and Mental Health Services Administration. Retrieved from https://www.samhsa.gov/data/.

[63] Weinberger AH, Gbedemah M, Martinez AM, Nash D, Galea S, Goodwin RD (2018). Trends in depression prevalence in the USA from 2005 to 2015: widening disparities in vulnerable groups. Psychol Med.

[64] Shi P, Yang A, Zhao Q, Chen Z, Ren X, Dai Q (2021). A Hypothesis of gender differences in self-reporting symptom of depression: implications to solve under-diagnosis and under-treatment of depression in males. Front Psychiatry.

[65] Sigmon ST, Pells JJ, Boulard NE, Whitcomb-Smith S, Edenfield TM, Hermann BA, LaMattina SM, Schartel JG, Kubik E (2005). Gender differences in self-reports of depression: the response bias hypothesis revisited. Sex Roles.

[66] Adams LB, Baxter SLK, Lightfoot AF, Gottfredson N, Golin C, Jackson LC, Tabron J, Corbie-Smith G, Powell W (2021). Refining Black men’s depression measurement using participatory approaches: a concept mapping study. BMC Public Health.

[67] Hudson DL, Eaton J, Banks A, Sewell W, Neighbors H (2018). "Down in the sewers": perceptions of depression and depression care among African American men. Am J Mens Health.

[68] Chun A, Reinhardt JP, Ramirez M, Ellis JM, Silver S, Burack O, Eimicke JP, Cimarolli V, Teresi JA (2017). Depression recognition and capacity for self-report among ethnically diverse nursing homes residents: evidence of disparities in screening. J Clin Nurs.

[69] Dagher RK, Bruckheim HE, Colpe LJ, Edwards E, White DB (2021). Perinatal depression: challenges and opportunities. J Womens Health (Larchmt).

[70] Vellakkal S, Subramanian SV, Millett C, Basu S, Stuckler D, Ebrahim S (2013). Socioeconomic inequalities in non-communicable diseases prevalence in India: disparities between self-reported diagnoses and standardized measures. PLoS ONE.

[71] Substance Abuse and Mental Health Services Administration (SAMHSA). (2020). Key substance use and mental health indicators in the United States: Results from the 2019 National Survey on Drug Use and Health (HHS Publication No. PEP20–07–01–001, NSDUH Series H-55). Rockville, MD: Center for Behavioral Health Statistics and Quality, Substance Abuse and Mental Health Services Administration Retrieved from https://www.samhsagov/data/.

[72] 2019 NSDUH Detailed Tables, Section 11: Youth Mental Health Trend Tables - 11.1 to 11.14.

[73] 2019 NSDUH Detailed Tables, Section 10: Adult Mental Health Trend Tables - 10.1 to 10.43 [https://www.samhsa.gov/data/sites/default/files/reports/rpt29394/NSDUHDetailedTabs2019/NSDUHDetTabsSect10pe2019.htm]

[74] Stone DM, Simon TR, Fowler KA, Kegler SR, Yuan K, Holland KM, Ivey-Stephenson AZ, Crosby AE (2018). Vital signs: trends in state suicide rates - United States, 1999–2016 and circumstances contributing to suicide - 27 states, 2015. MMWR Morb Mortal Wkly Rep.

[75] Brown L, Tucker-Seeley R (2018). Commentary: will 'deaths of despair' among whites change how we talk about racial/ethnic health disparities?. Ethn Dis.

[76] Case A, Deaton A (2015). Rising morbidity and mortality in midlife among white non-Hispanic Americans in the 21st century. Proc Natl Acad Sci U S A.

[77] Case A, Deaton A (2017). Mortality and morbidity in the 21(st) century. Brookings Pap Econ Act.

[78] Bor J, Cohen GH, Galea S (2017). Population health in an era of rising income inequality: USA, 1980–2015. Lancet.

[79] Steelesmith DL, Fontanella CA, Campo JV, Bridge JA, Warren KL, Root ED (2019). Contextual factors associated with county-level suicide rates in the United States, 1999 to 2016. JAMA Netw Open.

[80] Stein EM, Gennuso KP, Ugboaja DC, Remington PL (2017). The epidemic of despair among white Americans: trends in the leading causes of premature death, 1999–2015. Am J Public Health.

[81] Minton J, Green M, McCartney G, Shaw R, Vanderbloemen L, Pickett K (2017). Two cheers for a small giant? why we need better ways of seeing data: a commentary on: ‘rising morbidity and mortality in midlife among White non-Hispanic Americans in the 21st century’. Int J Epidemiol.

[82] Brody DJ, Pratt LA, Hughes JP (2018). Prevalence of depression among adults aged 20 and over: United States, 2013–2016. NCHS Data Brief.

[83] Wetherall K, Daly M, Robb KA, Wood AM, O'Connor RC (2015). Explaining the income and suicidality relationship: income rank is more strongly associated with suicidal thoughts and attempts than income. Soc Psychiatry Psychiatr Epidemiol.

[84] Kim JL, Kim JM, Choi Y, Lee TH, Park EC (2016). Effect of socioeconomic status on the linkage between suicidal ideation and suicide attempts. Suicide Life Threat Behav.

[85] Machado DB, Rasella D, Dos Santos DN (2015). Impact of income inequality and other social determinants on suicide rate in Brazil. PLoS ONE.

[86] Knipe DW, Gunnell D, Pieris R, Priyadarshana C, Weerasinghe M, Pearson M, Jayamanne S, Dawson AH, Mohamed F, Gawarammana I (2017). Is socioeconomic position associated with risk of attempted suicide in rural Sri Lanka? a cross-sectional study of 165 000 individuals. BMJ Open.

[87] Knipe DW, Carroll R, Thomas KH, Pease A, Gunnell D, Metcalfe C (2015). Association of socio-economic position and suicide/attempted suicide in low and middle income countries in South and South-East Asia - a systematic review. BMC Public Health.

[88] Herne MA, Bartholomew ML, Weahkee RL (2014). Suicide mortality among American Indians and Alaska Natives, 1999–2009. Am J Public Health.

[89] Shiels MS, Chernyavskiy P, Anderson WF, Best AF, Haozous EA, Hartge P, Rosenberg PS, Thomas D, Freedman ND (2017). Berrington de Gonzalez A: trends in premature mortality in the USA by sex, race, and ethnicity from 1999 to 2014: an analysis of death certificate data. Lancet.

[90] Marrast L, Himmelstein DU, Woolhandler S (2016). Racial and ethnic disparities in mental health care for children and young adults: a national study. Int J Health Serv.

[91] Christensen H, Cuijpers P, Reynolds CF (2016). Changing the direction of suicide prevention research: a necessity for true population impact. JAMA Psychiat.

[92] Andrilla CHA, Patterson DG, Garberson LA, Coulthard C, Larson EH (2018). Geographic variation in the supply of selected behavioral health providers. Am J Prev Med.

[93] Bastiampillai T, Sharfstein SS, Allison S (2016). Increase in US suicide rates and the critical decline in psychiatric beds. JAMA.

[94] Allison S, Bastiampillai T, Licinio J, Fuller DA, Bidargaddi N, Sharfstein SS (2018). When should governments increase the supply of psychiatric beds?. Mol Psychiatry.

[95] Hung P, Busch SH, Shih YW, McGregor AJ, Wang S (2020). Changes in community mental health services availability and suicide mortality in the US: a retrospective study. BMC Psychiatry.

[96] While D, Bickley H, Roscoe A, Windfuhr K, Rahman S, Shaw J, Appleby L, Kapur N (2012). Implementation of mental health service recommendations in England and Wales and suicide rates, 1997–2006: a cross-sectional and before-and-after observational study. Lancet.

[97] Zalsman G, Hawton K, Wasserman D, van Heeringen K, Arensman E, Sarchiapone M, Carli V, Hoschl C, Barzilay R, Balazs J (2016). Suicide prevention strategies revisited: 10-year systematic review. Lancet Psychiatry.

[98] Courtet P, Lopez-Castroman J (2017). Antidepressants and suicide risk in depression. World Psychiatry.

[99] Fornaro M, Anastasia A, Valchera A, Carano A, Orsolini L, Vellante F, Rapini G, Olivieri L, Di Natale S, Perna G (2019). The FDA "Black Box" warning on antidepressant suicide risk in young adults: more harm than benefits?. Front Psychiatry.

[100] Gusmao R, Quintao S, McDaid D, Arensman E, Van Audenhove C, Coffey C, Varnik A, Varnik P, Coyne J, Hegerl U (2013). Antidepressant utilization and suicide in Europe: an ecological multi-national study. PLoS ONE.

[101] Brenner E: The Crisis of Youth Mental Health. In: Stanford Social Innovation Review, Spring 2019. https://ssir.org/articles/entry/the_crisis_of_youth_mental_health.

[102] WISQARS™ Fatal Injury Data: Leading Causes of Death Reports, 1981 - 2018 [https://www.webappa.cdc.gov/sasweb/ncipc/leadcause.html]

[103] Franklin JC, Ribeiro JD, Fox KR, Bentley KH, Kleiman EM, Huang X, Musacchio KM, Jaroszewski AC, Chang BP, Nock MK (2017). Risk factors for suicidal thoughts and behaviors: a meta-analysis of 50 years of research. Psychol Bull.

[104] Carey B: How Suicide Quietly Morphed Into a Public Health Crisis. In: The New York Times, . June 8, 2018, https://www.nytimes.com/2018/06/08/health/suicide-spade-bordain-cdc.html.

[105] Foundation Health Measures, Disparities [https://www.healthypeople.gov/2020/about/foundation-health-measures/Disparities]

[106] Yip PS, Caine E, Yousuf S, Chang SS, Wu KC, Chen YY (2012). Means restriction for suicide prevention. Lancet.

[107] Kellermann AL, Rivara FP, Somes G, Reay DT, Francisco J, Banton JG, Prodzinski J, Fligner C, Hackman BB (1992). Suicide in the home in relation to gun ownership. N Engl J Med.

[108] Wiebe DJ (2003). Homicide and suicide risks associated with firearms in the home: a national case-control study. Ann Emerg Med.

[109] Anglemyer A, Horvath T, Rutherford G (2014). The accessibility of firearms and risk for suicide and homicide victimization among household members: a systematic review and meta-analysis. Ann Intern Med.

[110] Miller M, Barber C, Azrael D, Hemenway D, Molnar BE (2009). Recent psychopathology, suicidal thoughts and suicide attempts in households with and without firearms: findings from the National Comorbidity Study Replication. Inj Prev.

[111] Miller M, Barber C, White RA, Azrael D (2013). Firearms and suicide in the United States: is risk independent of underlying suicidal behavior?. Am J Epidemiol.

[112] Miller M, Lippmann SJ, Azrael D, Hemenway D (2007). Household firearm ownership and rates of suicide across the 50 United States. J Trauma.

[113] Kposowa A, Hamilton D, Wang K (2016). Impact of firearm availability and gun regulation on state suicide rates. Suicide Life Threat Behav.

[114] Mann JJ, Michel CA (2016). Prevention of firearm suicide in the United States: what works and what is possible. Am J Psychiatry.

[115] Opoliner A, Azrael D, Barber C, Fitzmaurice G, Miller M (2014). Explaining geographic patterns of suicide in the US: the role of firearms and antidepressants. Inj Epidemiol.

[116] Miller M, Hemenway D (2008). Guns and suicide in the United States. N Engl J Med.

[117] Simon OR, Swann AC, Powell KE, Potter LB, Kresnow MJ, O'Carroll PW (2001). Characteristics of impulsive suicide attempts and attempters. Suicide Life Threat Behav.

[118] Lewiecki EM, Miller SA (2013). Suicide, guns, and public policy. Am J Public Health.

[119] Kalesan B, Villarreal MD, Keyes KM, Galea S (2016). Gun ownership and social gun culture. Inj Prev.

[120] Studdert DM, Zhang Y, Swanson SA, Prince L, Rodden JA, Holsinger EE, Spittal MJ, Wintemute GJ, Miller M (2020). Handgun ownership and suicide in California. N Engl J Med.

[121] Gunter J: The silent epidemic of America’s problem with guns.

[122] Riddell CA, Harper S, Cerda M, Kaufman JS: Comparison of Rates of Firearm and Nonfirearm Homicide and Suicide in Black and White Non-Hispanic Men, by U.S. State. Ann Intern Med 2018, 168(10):712–720. https://www.mcgill.ca/newsroom/channels/news/suicide-and-homicide-rates-show-large-racial-disparities-across-us-states-286782. 10.7326/M17-2976 29710093

[123] Pew Research Center. America’s Complex Relationship With Guns [http://assets.pewresearch.org/wp-content/uploads/sites/3/2017/06/06151541/Guns-Report-FOR-WEBSITE-PDF-6-21.pdf]

[124] Party Identity in a Gun Cabinet [https://fivethirtyeight.blogs.nytimes.com/2012/12/18/in-gun-ownership-statistics-partisan-divide-is-sharp/]

[125] Chamard S: Correlates of Gun Ownership in Anchorage and the Mat-Su Borough. Alaska Justice Forum, 27(2)(Summer 2010):2–4

[126] Grinshteyn E, Hemenway D (2016). Violent death rates: the US compared with other high-income OECD countries, 2010. Am J Med.

[127] Duquet N, Van Alstein M (2015). Firearms and violent deaths in Europe.

[128] States with Weak Gun Laws and Higher Gun Ownership Lead Nation in Gun Suicides [http://www.vpc.org/press/states-with-weak-gun-laws-and-higher-gun-ownership-lead-nation-in-gun-suicides/]

[129] Fleegler EW, Lee LK, Monuteaux MC, Hemenway D, Mannix R (2013). Firearm legislation and firearm-related fatalities in the United States. JAMA Intern Med.

[130] Kposowa AJ (2013). Association of suicide rates, gun ownership, conservatism and individual suicide risk. Soc Psychiatry Psychiatr Epidemiol.

[131] Kaufman EJ, Morrison CN, Branas CC, Wiebe DJ (2018). State firearm laws and interstate firearm deaths from homicide and suicide in the United States: a cross-sectional analysis of data by county. JAMA Intern Med.

[132] Geier DA, Kern JK, Geier MR (2017). A longitudinal ecological study of household firearm ownership and firearm-related deaths in the United States from 1999 through 2014: a specific focus on gender, race, and geographic variables. Prev Med Rep.

[133] Ringgenberg W, Peek-Asa C, Donham K, Ramirez M (2018). Trends and characteristics of occupational suicide and homicide in farmers and agriculture workers, 1992–2010. J Rural Health.

[134] ‘I’m gonna lose everything’: A farm family struggles to recover after rising debt pushes a husband to suicide [https://www.washingtonpost.com/nation/2019/11/09/im-gonna-lose-everything/?arc404=true]

[135] Curtin SC, Warner M, Hedegaard H (2016). Increase in suicide in the United States, 1999–2014. NCHS Data Brief.

[136] Miller M, Azrael D, Hepburn L, Hemenway D, Lippmann SJ (2006). The association between changes in household firearm ownership and rates of suicide in the United States, 1981–2002. Inj Prev.

[137] US Gun Deaths by Year [https://www.gun-control.procon.org/view.resource.php?resourceID=006094]

[138] Fatal Injury Reports, National, Regional and State, 1981 - 2018 [https://www.webappa.cdc.gov/sasweb/ncipc/mortrate.html]

[139] It’s Time to Recognize Suicide as a Driver of Gun-Related Deaths [https://www.harvardpolitics.com/united-states/suicide-gun-related-deaths/]

[140] Anestis MD, Anestis JC (2015). Suicide rates and state laws regulating access and exposure to handguns. Am J Public Health.

[141] Luca M, Malhotra D, Poliquin C (2017). Handgun waiting periods reduce gun deaths. Proc Natl Acad Sci U S A.

[142] Crifasi CK, Meyers JS, Vernick JS, Webster DW (2015). Effects of changes in permit-to-purchase handgun laws in Connecticut and Missouri on suicide rates. Prev Med.

[143] Grossman DC, Mueller BA, Riedy C, Dowd MD, Villaveces A, Prodzinski J, Nakagawara J, Howard J, Thiersch N, Harruff R (2005). Gun storage practices and risk of youth suicide and unintentional firearm injuries. JAMA.

[144] Sarai SK, Abaid B, Lippmann S: Guns and Suicide: Are They Related? Prim Care Companion CNS Disord 2017, 19(6). 10.4088/PCC.17br02116 29272571

[145] Appelbaum PS, Swanson JW (2010). Law & psychiatry: gun laws and mental illness: how sensible are the current restrictions?. Psychiatr Serv.

[146] Webster DW, Vernick JS, Zeoli AM, Manganello JA (2004). Association between youth-focused firearm laws and youth suicides. JAMA.

[147] Cummings P, Grossman DC, Rivara FP, Koepsell TD (1997). State gun safe storage laws and child mortality due to firearms. JAMA.

[148] Gius M (2015). The impact of minimum age and child access prevention laws on firearm-related youth suicides and unintentional deaths. Soc Sci J.

[149] Parker K, Horowitz J, Igielnik R, Oliphant B, Brown A 2017 America's complex relationship with guns: An in-depth look at the attitudes and experiences of US adults: Pew Research Center

[150] Butterworth SE, Anestis MD: Political beliefs, region of residence, and openness to firearm means safety measures to prevent suicide. Arch Suicide Res 2018:1–32 10.1080/13811118.2018.1486250 29952717

[151] Kivisto AJ, Phalen PL (2018). Effects of risk-based firearm seizure laws in connecticut and Indiana on suicide rates, 1981–2015. Psychiatr Serv..

[152] Betz ME, Wintemute GJ (2015). Physician counseling on firearm safety: a new kind of cultural competence. JAMA.

[153] Runyan CW, Becker A, Brandspigel S, Barber C, Trudeau A, Novins D (2016). Lethal means counseling for parents of youth seeking emergency care for suicidality. West J Emerg Med.

[154] Vriniotis M, Barber C, Frank E, Demicco R (2015). New hampshire firearm safety c: a suicide prevention campaign for firearm dealers in New Hampshire. Suicide Life Threat Behav.

[155] Gun Shop Project. Suicide Prevention: A Role for Firearm Dealers and Range Owners [https://www.hsph.harvard.edu/means-matter/gun-shop-project/]

[156] Swanson JW, Norko MA, Lin H-J, Alanis-Hirsch K, Frisman LK, Baranoski MV, Easter MM, Robertson AG, Swartz MS, Bonnie RJ (2017). Implementation and effectiveness of connecticut's risk-based gun removal law: does it prevent suicides. Law & Contemp Probs.

[157] Rodriguez Andres A, Hempstead K (2011). Gun control and suicide: the impact of state firearm regulations in the United States, 1995–2004. Health Policy.

[158] PUBLIC LAW 104–208—SEPT. 30, 1996 [https://www.gpo.gov/fdsys/pkg/PLAW-104publ208/pdf/PLAW-104publ208.pdf]

[159] Jamieson C: Gun violence research: History of the federal funding freeze. Psychological Science Agenda, February 2013.

[160] Hemenway D (2017). Fight the silencing of gun research. Nature.

[161] Wadman M (2013). Firearms research: the gun fighter. Nature.

[162] Rubin R (2016). Tale of 2 Agencies: CDC avoids gun violence research but NIH funds it. JAMA.

[163] Caswell W: Gun Violence reframed as a Public Health issue. Modern Health Talk, 03/05/2018, http://www.mhealthtalk.com/guns/.

[164] Stark DE, Shah NH (2017). Funding and publication of research on gun violence and other leading causes of death. JAMA.

[165] Kristof N: How to Reduce Shootings. New York Times, May 18, 2018, https://www.nytimes.com/interactive/2017/11/06/opinion/how-to-reduce-shootings.html.

[166] Americans Overwhelmingly See Gun Violence as a Public Health Issue; They Want Congress to Act and CDC to Conduct Research. American Psychiatric Association (APA) Public Opinion Poll, May 07, 2018, https://www.psychiatry.org/newsroom/news-releases/americans-overwhelmingly-see-gun-violence-as-a-public-health-issue-they-want-congress-to-act-and-cdc-to-conduct-research.

[167] World Health Organization. WHO Director-General's opening remarks at the media briefing on COVID-19 - 11 March 2020 [https://www.who.int/dg/speeches/detail/who-director-general-s-opening-remarks-at-the-media-briefing-on-covid-19---11-march-2020]

[168] Turecki G, Brent DA (2016). Suicide and suicidal behaviour. Lancet.

[169] America on edge: Covid lockdowns, protests and election strife led to record gun sales [https://www.washingtonpost.com/national/record-gun-sales-us-2020/2021/01/18/d25e8616-55a9-11eb-a931-5b162d0d033d_story.html]

[170] Taking Stock of Record-Setting 2020 Firearm Year [https://www.nssf.org/articles/taking-stock-of-record-setting-2020-firearm-year/]

[171] Pandemic And Protests Spark Record Gun Sales [https://www.npr.org/2020/07/16/891608244/protests-and-pandemic-spark-record-gun-sales]

[172] Reger MA, Stanley IH, Joiner TE (2020). Suicide mortality and coronavirus disease 2019-a perfect storm?. JAMA Psychiatry.

[173] Gunnell D, Appleby L, Arensman E, Hawton K, John A, Kapur N, Khan M, O'Connor RC, Pirkis J (2020). Collaboration C-SPR: Suicide risk and prevention during the COVID-19 pandemic. Lancet Psychiatry..

[174] Sher L (2020). The impact of the COVID-19 pandemic on suicide rates. QJM.

[175] US suicides dropped last year, defying pandemic expectations [https://www.apnews.com/article/pandemics-suicide-prevention-coronavirus-pandemic-d8d9168403baa6660e5125c040b2ae81?]

[176] Ahmad FB, Anderson RN (2021). the leading causes of death in the US for 2020. JAMA.

[177] Pirkis J, John A, Shin S, DelPozo-Banos M, Arya V, Analuisa-Aguilar P, Appleby L, Arensman E, Bantjes J, Baran A (2021). Suicide trends in the early months of the COVID-19 pandemic: an interrupted time-series analysis of preliminary data from 21 countries. Lancet Psychiatry.

[178] Banerjee D, Kosagisharaf JR, Sathyanarayana Rao TS (2021). 'The dual pandemic' of suicide and COVID-19: a biopsychosocial narrative of risks and prevention. Psychiatry Res.

[179] https://www.usnews.com/news/health-news/articles/2022-03-23/pandemics-long-term-effects-on-suicide-still-in-question.

[180] Bray MJC, Daneshvari NO, Radhakrishnan I, Cubbage J, Eagle M, Southall P, Nestadt PS (2021). Racial differences in statewide suicide mortality trends in Maryland during the coronavirus disease 2019 (COVID-19) pandemic. JAMA Psychiat.

[181] Suicides Rise in Black Population During COVID-19 Pandemic [https://www.hopkinsmedicine.org/news/articles/suicides-rise-in-black-population-during-covid-19-pandemic]

[182] Yard E, Radhakrishnan L, Ballesteros MF, Sheppard M, Gates A, Stein Z, Hartnett K, Kite-Powell A, Rodgers L, Adjemian J et al: Emergency Department Visits for Suspected Suicide Attempts Among Persons Aged 12–25 Years Before and During the COVID-19 Pandemic — United States, January 2019–May 2021. MMWR Morb Mortal Wkly Rep 2021, ePub: 11 June 2021 10.15585/mmwr.mm7024e1 10.15585/mmwr.mm7024e1 PMC8220953 34138833

[183] Gracia R, Pamias M, Mortier P, Alonso J, Pérez V, Palao D (2021). Is the COVID-19 pandemic a risk factor for suicide attempts in adolescent girls?. J Affect Disord.

[184] John A, Eyles E, Webb RT, Okolie C, Schmidt L, Arensman E, Hawton K, O'Connor RC, Kapur N, Moran P (2020). The impact of the COVID-19 pandemic on self-harm and suicidal behaviour: update of living systematic review. F1000Res.

[185] The Racial Impacts of COVID-19 [https://www.embracerace.org/resources/disproportionate-racial-impacts-of-covid#asianamericans]

[186] COVID-19 in Racial and Ethnic Minority Groups [https://www.cdc.gov/coronavirus/2019-ncov/need-extra-precautions/racial-ethnic-minorities.html] 10.1007/s40615-021-01170-w PMC8513546 34647273

[187] Chan SM, Chiu FK, Lam CW, Leung PY, Conwell Y (2006). Elderly suicide and the 2003 SARS epidemic in Hong Kong. Int J Geriatr Psychiatry.

[188] Cheung YT, Chau PH, Yip PS (2008). A revisit on older adults suicides and Severe Acute Respiratory Syndrome (SARS) epidemic in Hong Kong. Int J Geriatr Psychiatry.

[189] The Covid-19 crisis too few are talking about: health care workers’ mental health [https://www.statnews.com/2020/04/03/the-covid-19-crisis-too-few-are-talking-about-health-care-workers-mental-health/]

[190] Top E.R. Doctor Who Treated Virus Patients Dies by Suicide [https://www.nytimes.com/2020/04/27/nyregion/new-york-city-doctor-suicide-coronavirus.html]

[191] Guns and Suicide [https://www.jec.senate.gov/public/_cache/files/15702336-e9b5-4258-bf3d-c53b407cf550/jec2019-gunsandsuicide-final.pdf]

[192] OECD 2021 Suicide rates (indicator). https://www.oecd-ilibrary.org/content/data/a82f3459-en doi: 10.1787/a82f3459-en Accessed on 30 Jun 2021

[193] Caine ED (2019). Seeking to prevent suicide at the edge of the ledge. Ann Intern Med.

[194] Caine ED (2019). Building the foundation for comprehensive suicide prevention - based on intention and planning in a social-ecological context. Epidemiol Psychiatr Sci.

[195] Gunnell D, Knipe D, Chang SS, Pearson M, Konradsen F, Lee WJ, Eddleston M (2017). Prevention of suicide with regulations aimed at restricting access to highly hazardous pesticides: a systematic review of the international evidence. Lancet Glob Health.

